# Assessing Strengths and Limitations of Magnetoencephalography Source Imaging With Intracerebral EEG

**DOI:** 10.1002/advs.76365

**Published:** 2026-07-08

**Authors:** Jawata Afnan, Maria Fratello, Francesca Bonini, Samuel Medina Villalon, Zhengchen Cai, Jean‐Marc Lina, Jean‐Michel Badier, Fabrice Bartolomei, Jean Gotman, Christian G. Bénar, Christophe Grova

**Affiliations:** ^1^ Multimodal Functional Imaging Lab Biomedical Engineering Department McGill University Montréal Québec Canada; ^2^ Integrated Program in Neuroscience McGill University Montréal Québec Canada; ^3^ Montreal Neurological Institute Department of Neurology and Neurosurgery McGill University Montréal Québec Canada; ^4^ Aix Marseille University INSERM INS Inst Neurosci Syst Marseille France; ^5^ APHM Timone Hospital Epileptology and Cerebral Rhythmology Marseille France; ^6^ Physnum Team Centre De Recherches Mathématiques Montréal Québec Canada; ^7^ Electrical Engineering Department École De Technologie Supérieure Montréal Québec Canada; ^8^ Multimodal Functional Imaging Lab Department of Physics and Concordia School of Health Concordia University Montréal Québec Canada

**Keywords:** deep brain imaging, epilepsy, functional connectivity, MEG source imaging, resting state

## Abstract

Due to the ill‐posed nature of source imaging, EEG/MEG source localization remains challenging, particularly for localizing deep brain activity and resting‐state signals with low signal‐to‐noise ratios. Functional connectivity estimated by EEG/MEG is further affected by source‐leakage, a spatial blurring effect that complicates interpretation. Validation is therefore essential before applying EEG/MEG source imaging in clinical contexts. Using simultaneous MEG and stereotactic EEG (SEEG) recordings from patients with focal epilepsy, we present a comprehensive validation of MEG source imaging of epileptic discharges, resting‐state oscillations, and connectivity. Virtual SEEG was computed from MEG source maps to enable direct quantitative comparison with in situ SEEG. Using the Maximum Entropy on the Mean method, MEG localized the generators of epileptic spikes with a median error of 15 ± 12 mm, with deep generators showing larger errors. MEG‐derived resting‐state power showed significant spatial correlations with SEEG, with frequency‐specific correspondence, stronger in the alpha and beta bands than in the theta band. MEG functional connectomes estimated using leakage‐corrected amplitude envelope correlation aligned relatively well with SEEG, whereas connectomes from the leakage‐corrected phase‐based metric were inaccurate. These findings delineate conditions under which MEG source imaging reliably captures epileptic and resting‐state activity, even with low signal‐to‐noise ratios, and conditions when it is unreliable.

## Graphical abstract

Simultaneous MEG and stereotactic EEG (SEEG) recordings provide a direct validation framework for MEG source imaging in focal epilepsy. Virtual SEEG signals derived from MEG reconstructions reveal significant agreement with intracranial measures of spike localization, resting‐state oscillations, and functional connectivity, while also identifying conditions under which MEG‐SEEG correspondence is strong or limited.

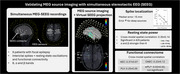

## 1 Introduction

Electro‐/Magneto‐EncephaloGraphy (EEG/MEG) is a widely used non‐invasive method to measure neuronal activity in healthy subjects [1] and pathological conditions [2]. The high temporal resolution of EEG/MEG allows studying brain activity during well‐controlled tasks or spontaneous abnormal discharges in epilepsy [3], as well as during the resting state, the analysis of which can be used to characterize normal [4] or pathological brain activity [5]. However, EEG/MEG has limited spatial resolution because it involves scalp recordings, and estimating the neuronal generators from these recordings—source imaging—requires solving an ill‐posed inverse problem [6]. Moreover, localizing deep generators using EEG/MEG is even more challenging because signals originating from these sources are associated with low signal‐to‐noise ratio (SNR) at the surface, due to the distance and the spatial configuration of subcortical structures [7, 8]. EEG/MEG source imaging is also affected by source leakage, defined as the influence of a source on the estimation of the generators within its neighborhood [9, 10]. Source leakage introduces spurious false positives in EEG/MEG‐derived connectivity measures. It also leads to spurious connectivity estimates that are insensitive to zero‐lag synchronization [9]. Thus, the validation of EEG/MEG source imaging is critical, especially in the context of localizing low SNR data, such as for EEG/MEG sources in deep structure or ongoing resting‐state data. This challenging source localization task is especially critical in clinical applications, such as pre‐surgical planning for epilepsy [8] and the study of the mesio‐temporal structures, including the hippocampus, often implicated in neurodegenerative diseases such as Alzheimer's disease and dementia, and in mesial temporal epilepsy.

Due to the frequent lack of ground truth, validation of EEG/MEG source imaging techniques often relies on numeric simulations. Realistic simulations, for instance, combining artificially simulated generators and real EEG/MEG background, are useful when validating source imaging for epileptic discharges [11] or in connectivity studies [9, 12]. Neuronal computational models using biophysical/physiological generative models to simulate realistic data are also of interest for validating source imaging methods [13, 14, 15]. While simulations of pathological discharges mimicking epileptic activity are feasible [16], simulating realistic background resting‐state activity is considerably more difficult. Such activity can provide meaningful insights into ongoing large‐scale network interactions [13, 17], but simulating resting‐state EEG/MEG data still lacks sufficient realism for validation purposes.

A few studies have attempted to validate EEG/MEG‐derived connectivity by comparing it with functional MRI (fMRI) connectivity [10, 18, 19]. Although such multimodal comparisons can be informative at a global level, EEG/MEG and fMRI are based on fundamentally different physiological mechanisms (electrophysiological signals vs. hemodynamic responses), making direct comparisons and coupling modeling challenging [18].

The gold standard for validating EEG/MEG source imaging is intracerebral EEG or stereotactic EEG (SEEG), an invasive technique commonly used in the pre‐surgical evaluation of drug‐resistant epilepsy. SEEG can record in situ brain activity directly from brain tissue, including deep structures, with high SNR and high spatial and temporal resolution [20]. SEEG measurements are also negligibly affected by volume conduction [21]. However, SEEG requires a surgical procedure to implant the electrodes and has limited spatial coverage. Atlases of intracranial EEG (iEEG) have been developed by pooling iEEG data from different patients, but only including channels that were implanted in presumably healthy regions [22, 23]. Such atlases provide a unique opportunity to validate EEG/MEG source imaging at a group level. We previously leveraged the atlas of normal iEEG developed at the Montreal Neurological Institute (MNI) [22] to validate MEG source imaging of resting‐state oscillations [24] and connectivity patterns [25] for a group of healthy subjects. Although the iEEG atlas provided an excellent way to validate MEG source imaging at a whole‐brain level, several limitations were unavoidable, such as the heterogeneity of iEEG sampling across the brain, variability in the number of channels contributed by different patients, the non‐simultaneity of the MEG and SEEG recordings, and the fact that the presumably healthy regions of epileptic patients may be different from totally healthy brains [24, 25].

Simultaneous recording of EEG/MEG and SEEG provides the most reliable validation for EEG/MEG [26, 27, 28, 29, 30], although limited to the implanted brain regions. SEEG has been used to assess the detectability of epileptic abnormalities by simultaneously acquired scalp EEG/MEG [26, 28, 31]. Two recent studies provided evidence of detectability from deep subcortical structures by high‐density EEG [32] and MEG [28]. However, studies using simultaneous EEG/MEG and SEEG to investigate resting‐state oscillations are very few [32, 33], and connectivity was not investigated. In this study, we present a comprehensive validation of MEG source imaging of epileptic spikes, resting‐state oscillations, and resting‐state connectivity by comparing them with simultaneously acquired SEEG as the ground truth in nine patients with epilepsy.

## 2 Results

### 2.1 Analysis Pipeline

**FIGURE 1:**
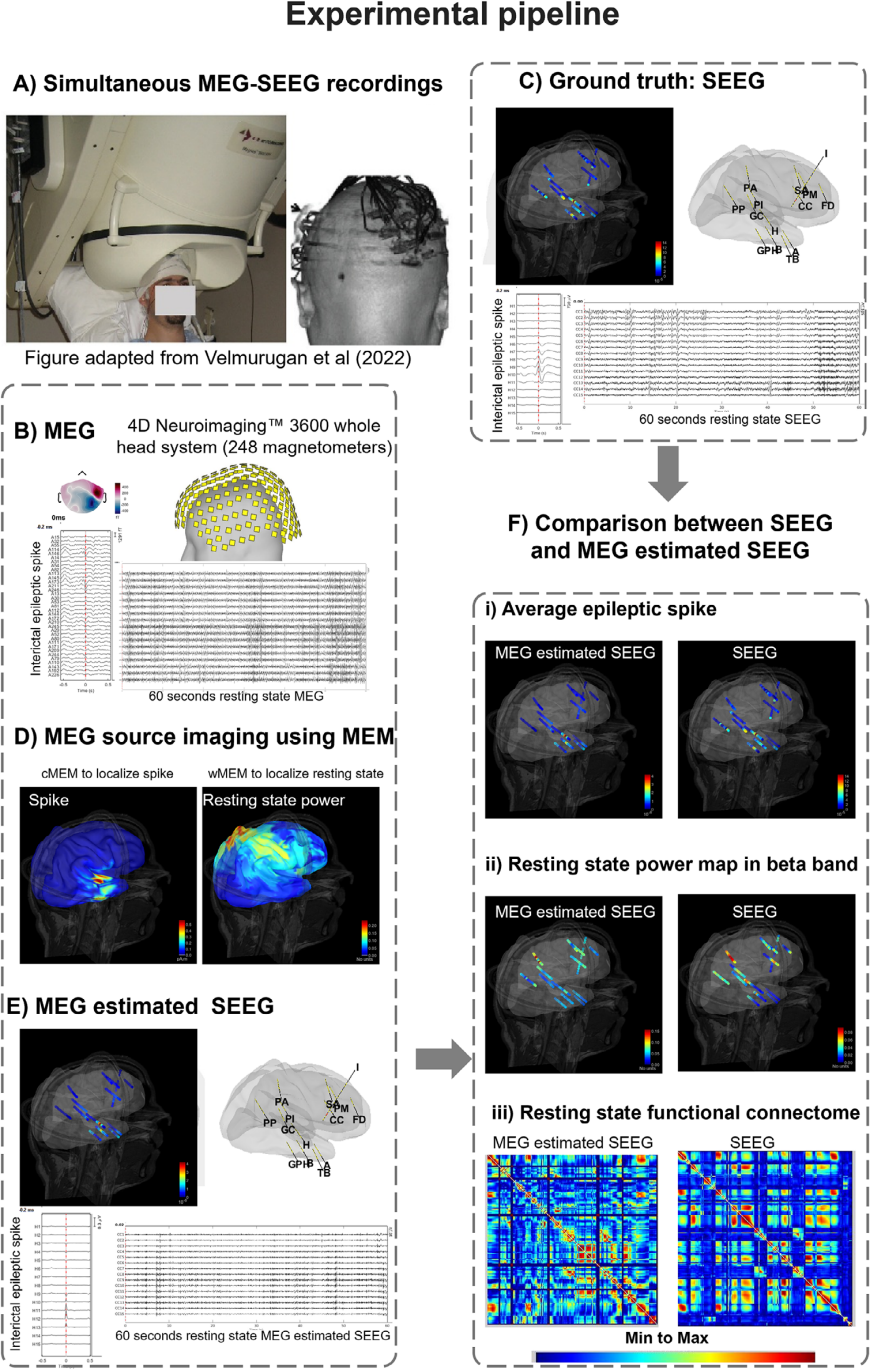
Analysis pipeline to validate epileptic spikes, resting state oscillation, and connectivity patterns estimated by MEG with simultaneous SEEG, as ground truth. (A) Simultaneous recordings of MEG and SEEG data from nine patients with focal epilepsy (adapted from Velmurugan et al., [34]). (B) MEG: Interictal epileptic spikes and 60 s of resting state data during wakefulness were marked in MEG in a dominant frequency band. (C) SEEG: Using the marking from MEG, interictal epileptic spikes and 60s of data were obtained from the simultaneously acquired SEEG, which is considered the ground truth. (D) MEG source imaging: The MEG inverse problem was solved using coherent Maximum Entropy on the Mean (cMEM) for spikes and the wavelet MEM (wMEM) for resting state activity. (E) MEG estimated SEEG: The MEG source maps were converted to virtual SEEG potentials for each SEEG channel location. (F) Comparison between MEG estimated SEEG and SEEG: The MEG estimated SEEG potentials were quantitatively compared with SEEG in terms of (i) average epileptic spikes localization, (ii) resting state oscillatory power in the dominant frequency band, and (iii) resting state functional connectivity computed using Amplitude Envelope Correlation (AEC), Orthogonalized Amplitude Envelope Correlation (OAEC), Phase Locking Value (PLV), and Weighted Phase Lag Index (wPLI*).

The analysis pipeline is summarized in Figure 1. Simultaneous MEG and SEEG recordings from nine patients with focal epilepsy were analyzed (Figure 1A and Table 1). Interictal epileptic spikes and resting‐state segments were visually marked in MEG by the epileptologist F.B. (Figure 1B). 60 s of resting‐state data were selected to avoid epileptic discharges and to contain a clearly defined dominant frequency band. Among the nine patients, three had a dominant frequency in the theta band (4–7 Hz), three in the alpha band (8–13 Hz), and three in the beta band (13–30 Hz). These MEG‐based time markers were then used to extract the corresponding segments from the simultaneously acquired SEEG (Figure 1C).

**TABLE 1:** Patient summary.

ID	Sex	Age at epilepsy onset	Age at SEEG	Age at surgery	Engel score	Epilepsy type	Epilepsy type detail	Previous operation	Anatomical lesion	RF‐TC	Histopathology
P1	M	1	32	n/a	n/a	Multilobar	Insulo‐parieto‐premotor	No	No	Yes	n/a
P2	M	2	19	20	Ia	Frontal	FLE	No	No	Yes	Non‐specific
P3	M	3	40	42	II	Temporal	MLTLE	Yes	Yes	Yes	Non‐specific
P4	M	11	33	n/a	n/a	Temporal	Bilateral TLE	No	No	Yes	n/a
P5	F	13	22	23	III	Temporal	Right LTLE	No	No	Yes	Non‐specific
P6	M	39	56	58	Ia	Multilobar	Left T+TLE	No	Yes	Yes	Hippocampal sclerosis
P7	F	11	48	n/a	I(RF‐TC)	Temporal	MTLE	No	Yes	Yes	n/a
P8	M	28	39	42	Ia	Frontal	Prefrontal	No	Yes	Yes	Gliosis
P9	M	2	21	22	III	Temporal	LTLE	Yes	Yes	Yes	Non‐specific

Abbreviation: FLE: Frontal Lobe Epilepsy, MLTLE: Mesio‐Lateral Temporal Lobe Epilepsy, TLE: Temporal Lobe Epilepsy, LTLE: Lateral Temporal Lobe Epilepsy, MTLE: Mesial Temporal Lobe Epilepsy, RF‐TC: Radiofrequency Thermocoagulation.

MEG source imaging was performed using coherent Maximum Entropy on the Mean (cMEM) [11, 14] for spike localization and wavelet‐MEM (wMEM) for resting‐state activity [24, 35] (Figure 1D). The resulting cortical source maps were projected onto the SEEG channel locations to generate virtual SEEG signals (Figure 1E), as in Grova et al. [36], by applying a SEEG forward model to map MEG sources to intracranial locations. This enabled direct quantitative comparisons between MEG‐derived virtual SEEG and actual SEEG signals [36, 37]. Finally, we compared the two modalities for spike localization (Figure 1F‐i), resting‐state oscillatory power (Figure 1F‐ii), and functional connectivity (Figure 1F‐iii) within the dominant frequency band. Functional connectivity was assessed using a widely‐used amplitude‐based metric—the amplitude envelope correlation (*AEC*) [10] and a phase‐based metric, the phase locking value (*PLV*) [38, 39]. Additionally, we considered two metrics that remove zero‐lag connectivity: orthogonalized AEC (*OAEC*) [40] and a modified version of the weighted phase lag index (*wPLI*) [41], modified to consider only the phase information and evaluated in [25].

### 2.2 Example patient P1

**FIGURE 2:**
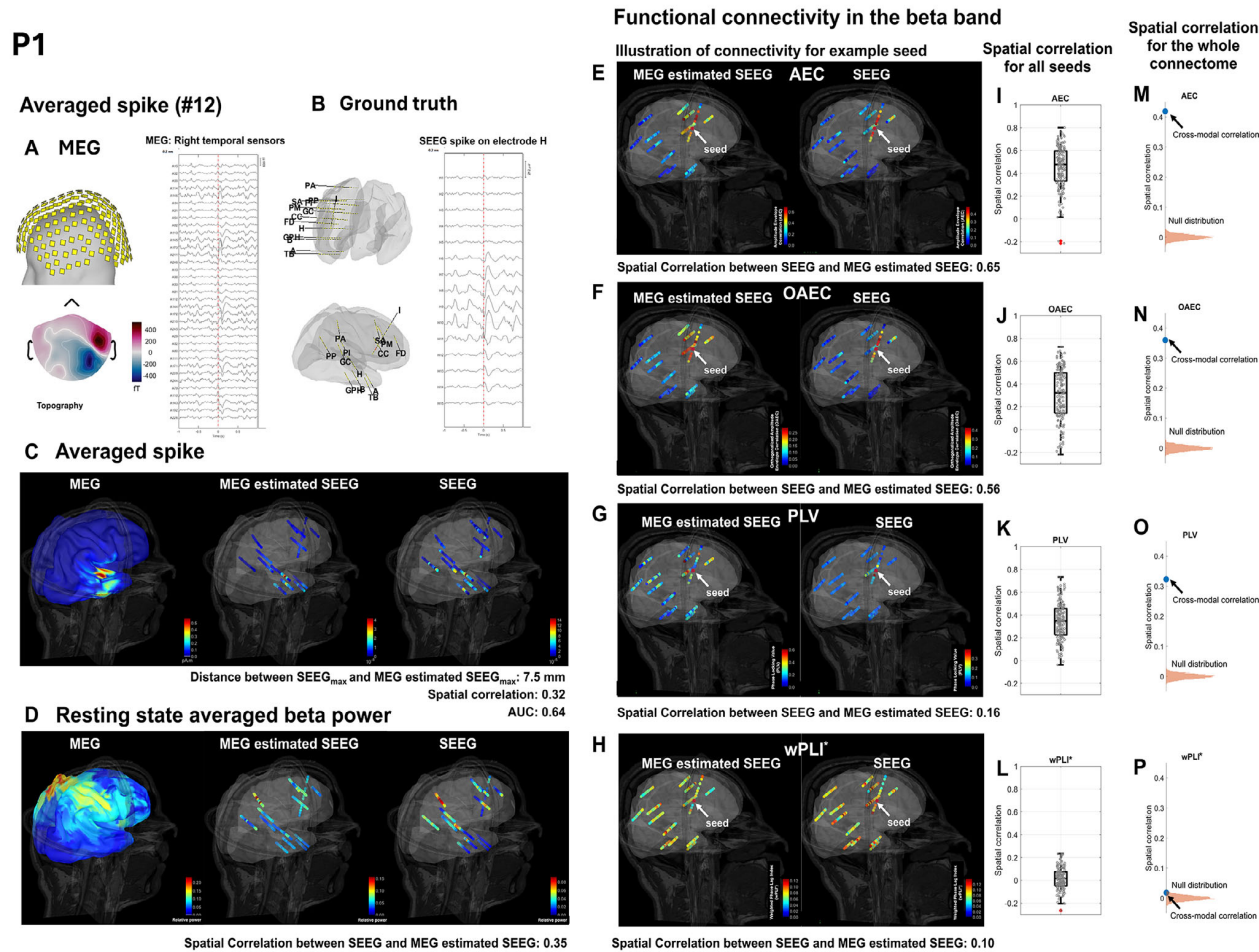
Validation of MEG source imaging with simultaneous SEEG for an example patient 1 (P1). (A) Average of 12 spikes in MEG displayed for right temporal sensors and topography. (B) Ground truth: The SEEG implantation on the right hemisphere and the average of 12 SEEG spikes shown on electrode H (Hippocampus). (C) The left panel shows the MEG source reconstructed map at the peak of the averaged spike. The right panel shows the activity of SEEG contacts at the peak of the averaged spike. The middle panel shows the MEG estimated SEEG map. Comparison between SEEG and MEG‐estimated SEEG at the time of the spike‐peak: Euclidean distance between maxima Dmin (7.5 mm), spatial correlation (0.32), and AUC (0.64). (D) Analysis of resting state power in the beta band: average beta band power is shown for the MEG data reconstructed on the cortical surface (left panel), MEG estimated SEEG (middle panel), and actual SEEG (right panel): spatial cross‐modal correlation between SEEG and MEG‐estimated SEEG (0.35). (E‐H) Functional connectivity in beta band: *Seed‐based connectivity*: The functional connectivity for an example seed (white arrow) is shown for SEEG and MEG estimated SEEG computed using (E) Amplitude Envelope Correlation (AEC), (F) Orthogonalized Amplitude Envelope Correlation (OAEC), (G) Phase Locking Value (PLV), and (H) weighted Phase Lag Index (wPLI*). For each connectivity metric, the spatial correlation between SEEG and MEG estimated SEEG maps computed for this example seed is displayed‐ AEC: 0.65, OAEC: 0.56, PLV: 0.16, and wPLI*: 0.10. The spatial correlations between SEEG and MEG estimated SEEG for all seeds are summarized as boxplot distributions for (I) AEC, (J) OAEC, (K) PLV, and (L) wPLI*. Outliers are shown as red dots. (M–P) *Whole connectome analysis*: The spatial correlation between the entire SEEG connectome and the MEG estimated connectomes is shown for AEC: 0.41 (M), OAEC: 0.36 (N), PLV: 0.32 (O), and wPLI*(0.02) (P). For each metric, the histogram of the null distribution is shown, and the actual spatial correlation value is shown as a blue dot. The null distribution was derived by 5000 spatial permutations of the MEG estimated SEEG contact labels.

Figure 2 illustrates the comparison pipeline for an example patient (P1) with right SEEG implantation.

For P1 with multilobar epilepsy, twelve spikes with similar right temporal topography were marked in MEG and averaged (Figure 2A). Using the same markers, spikes were extracted from simultaneous SEEG, showing maximal spike activity in the right superior temporal sulcus‐posterior at electrode H, contact H10. An average reference montage was used for the analysis of spike and resting‐state power.

The MEG‐reconstructed cMEM source map on the cortical surface exhibited right temporal activity (Figure 2C, left column). The corresponding MEG‐estimated SEEG map (Figure 2C, middle) showed maximum activity in the right superior temporal sulcus‐posterior. The distance between the SEEG and MEG‐estimated SEEG maxima (*Dmin*) was 7.5 mm (Figure 2C, middle and right). The spatial correlation between SEEG and MEG‐estimated SEEG at the spike‐peak was 0.32. This correlation was statistically significant, exceeding the 95% range of the null distribution derived by 5000 spatial permutations of the MEG‐estimated SEEG contact labels (see Materials and Methods). For P1, the *AUC* at the spike‐peak was 0.64, indicating a good spatial correspondence with some spatial overlap between the extents of the two maps, aligned with our previous results using non‐simultaneous MEG/SEEG comparison [37].

#### 2.2.1 Resting‐State Power in the Beta Band

For P1, the resting‐state 60 s segment was dominated by beta‐band oscillations. The MEG‐reconstructed average beta power on the cortical surface showed high activity in central regions, with greater intensity in the left hemisphere compared to the right (Figure 2D, left column). However, since electrodes were not implanted on the left, beta localization could not be validated. On the right hemisphere, the cross‐modal spatial correlation of beta maps was 0.35 and statistically significant (Figure 2D, middle and right columns).

#### 2.2.2 Resting‐State Functional Connectivity in the Beta Band

Functional connectivity during resting‐state was computed between all channel pairs in SEEG and in MEG‐estimated SEEG using a bipolar montage (see Materials and Methods). P1 had 162 bipolar channels, resulting in 13,041 channel pairs. We computed the cross‐modal correlation between SEEG and MEG‐estimated SEEG connectivity at two levels: the seed level, where cross‐modal correlation was calculated for each seed, yielding 162 correlation values (see boxplot distribution in Figure 2I–L), and the whole‐connectome level, where the full connectivity matrices were compared, yielding a single cross‐modal correlation value. *Seed‐based connectivity*: Figure 2E–H shows connectivity from a representative seed (white arrow) to all other contacts, using four metrics: *AEC*, *OAEC*, *PLV*, and wPLI*. For this example seed, the spatial correlation between SEEG and MEG‐estimated SEEG connectivity was‐ *AEC*:0.65, *OAEC*:0.56, *PLV*:0.16, and wPLI*:0.10.

We also computed spatial correlations between SEEG and MEG‐estimated SEEG connectivity between each seed contact and all other contacts. The resulting distributions are summarized in Figure 2I–L, exhibiting a higher average spatial correlation for *AEC* than for *OAEC* (*AEC*:0.48±0.20; *OAEC*:0.32±0.26). Spatial correlations for *PLV* were 0.35±0.18, whereas wPLI* correlations were very low overall (0.02±0.09).

We finally computed the spatial correlation between SEEG and MEG‐estimated SEEG for the entire connectome (Figure 2M–P). Significant spatial correlations were found for *AEC* (0.41), *OAEC* (0.36), and *PLV* (0.32), all exceeding the 95% range of the null distribution. The correlation for wPLI* was 0.02 and not statistically significant when compared to the null distribution.

### 2.3 Example patient P9

**FIGURE 3:**
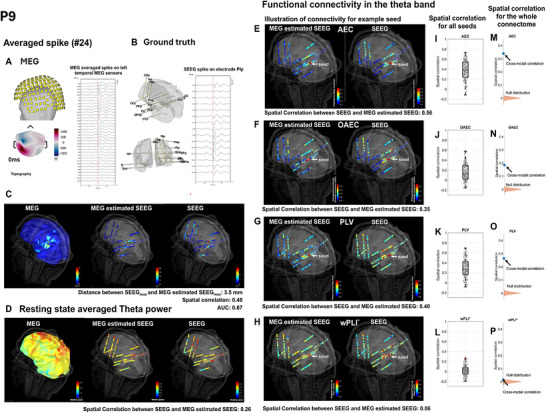
Validation of MEG source imaging with simultaneous SEEG for P9. (A) Average of 24 spikes in MEG shown for left parietal sensors and topography. (B) Ground truth: The SEEG implantation and the average of 24 SEEG spikes are shown. (C) At the peak of the averaged spike, the left panel shows the MEG source reconstructed map, the right panel shows the activity of SEEG contacts, and the middle panel shows the MEG estimated SEEG map: Euclidean distance between maxima Dmin (3.5 mm), spatial correlation (0.48), and AUC (0.67). (D) Analysis of resting state power in theta band: average theta band power is shown for the MEG reconstructed on the cortical surface (left panel), MEG estimated SEEG (middle panel), and actual SEEG (right panel): cross‐modal spatial correlation between SEEG and MEG‐estimated SEEG was 0.26. (E–H) Functional connectivity in theta band: The functional connectivity for an example seed (between the seed and all channels) is shown for SEEG and MEG estimated SEEG computed using (E) Amplitude Envelope Correlation (AEC): 0.56, (F) Orthogonalized Amplitude Envelope Correlation (OAEC): 0.35, (G) Phase Locking Value (PLV): 0.40 and (H) weighted Phase Lag Index (wPLI*): 0.06. For each connectivity metric, the spatial correlation between SEEG and MEG estimated SEEG maps computed for this example seed is displayed. The spatial correlations between SEEG and MEG estimated SEEG for all seeds are summarized as boxplot distributions for (I) AEC, (J) OAEC, (K) PLV, and (L) wPLI*. Outliers are shown as red dots. (M–P) *Whole connectome analysis*: The spatial correlation between the entire SEEG connectome and the MEG estimated connectomes is shown for AEC: 0.33 (M), OAEC: 0.18 (N), PLV: 0.26 (O), and wPLI*(0.02) (P). For each metric, the histogram of the null distribution is shown, and the actual spatial correlation value is shown as a blue dot. The null distribution was derived by 5000 spatial permutations of the MEG estimated SEEG contact labels.

Patient 9 (P9) provides another example where MEG accurately localized the average interictal spike with an error of 3.5 mm (Figure 3A–C). The SEEG spike‐peak was in the left postcentral sulcus at contact PIp11, while the MEG‐reconstructed SEEG peak was localized at the adjacent contact, PIp12. The spatial correlation between SEEG and MEG‐estimated SEEG spike maps was 0.48 (statistically significant) (Figure 3A–C). The *AUC* at the spike‐peak was 0.67, suggesting good spatial overlap. Resting‐state activity was dominated by theta oscillations. The MEG‐reconstructed average theta power displayed widespread cortical activity. The spatial correlation between SEEG and MEG‐estimated SEEG theta was 0.26 (Figure 3D) (statistically significant).

Resting‐state functional connectivity in theta band: *Seed‐based connectivity*: Spatial correlations between SEEG and MEG‐estimated SEEG across all seeds were as follows: *AEC*(0.38±0.24), *OAEC*(0.13±0.20), *PLV*(0.26±0.18), and wPLI*(0.02±0.09) (Figure 3I–L). *Whole connectome*: Spatial correlations between SEEG and MEG‐estimated SEEG mirrored the trends observed in P1. Spatial correlations were found significant for *AEC*(0.33), *OAEC*(0.18), and *PLV*(0.26) and not significant for wPLI* (0.02) (Figure 3M–P).

### 2.4 Example patient P4

**FIGURE 4:**
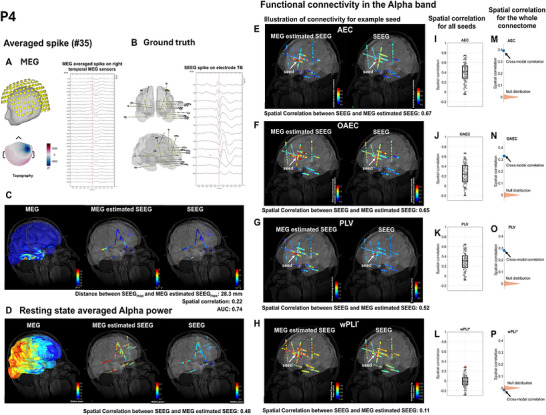
Validation of MEG source imaging with simultaneous SEEG for P4. (A) Average of 35 spikes in MEG shown with topography. (B) Ground truth: The SEEG implantation and the average of 35 spikes are shown. (C) At the peak of the averaged spike, the left panel shows the MEG source reconstructed map, the right panel shows the activity of SEEG contacts, and the middle panel shows the MEG estimated SEEG map. (D) Analysis of resting state power in the alpha band: average alpha band power is shown for the MEG reconstructed on the cortical surface (left panel), MEG estimated SEEG (middle panel), and actual SEEG (right panel). (E–H) Functional connectivity in alpha band: The functional connectivity for an example seed (between the seed and all channels) is shown for SEEG and MEG estimated SEEG computed using (E) Amplitude Envelope Correlation (AEC), (F) Orthogonalized Amplitude Envelope Correlation (OAEC), (G) Phase Locking Value (PLV) and (H) weighted Phase Lag Index (wPLI*). For each connectivity metric, the spatial correlation between SEEG and MEG estimated SEEG maps computed for this example seed is displayed. The spatial correlations between SEEG and MEG estimated SEEG for all seeds are summarized as boxplot distributions for (I) AEC, (J) OAEC, (K) PLV, and (L) wPLI*. Outliers are shown as red dots. (M–P) *Whole connectome analysis*: The spatial correlation between the entire SEEG connectome and the MEG estimated connectomes is shown for AEC (M), OAEC (N), PLV (O), and wPLI* (P). For each metric, the histogram of the null distribution is shown, and the actual spatial correlation value is shown as a blue dot. The null distribution was derived by 5000 spatial permutations of the MEG estimated SEEG contact labels.

In the two previous examples, MEG localization of spikes originating from the superficial cortical region was very accurate. Conversely, in Patient 4 (P4), spikes were generated in a deep region (the highest SEEG‐averaged spike activity was in contact TB4, in the right para‐hippocampal cortex), and the MEG‐estimated SEEG peak was localized to a more superficial contact, TB12. Dmin was 28.3 mm, the spatial correlation between SEEG and MEG‐estimated SEEG was 0.22 (statistically significant), and the AUC of 0.74 suggested still excellent spatial correspondence, although the deep SEEG peak was missed. Indeed, in SEEG, the spike originated at the deepest contact, TB1, approximately 40 ms before TB4 (Figure 4B), and then propagated rapidly to more superficial contacts (including TB10‐TB12). The spread of the SEEG activity to more distant electrodes (OR1‐OR5) also showed good correspondence between SEEG and MEG‐estimated SEEG. The MEG sensors detected the spike only when the underlying generators involved multiple SEEG contacts, including deep and superficial sites.

Resting‐state activity was dominated by alpha oscillations. The MEG‐reconstructed alpha power on the cortical surface displayed typical posterior alpha activity. However, electrodes were not implanted in the occipital regions, preventing direct validation of MEG localization in that area. Still, a comparison between SEEG and MEG‐estimated SEEG was feasible in the frontal and temporal regions. The spatial correlation between these two was 0.48, statistically significant. Qualitative comparison (Figure 4D, middle and right columns) showed that, although the overall patterns were similar across the two modalities, SEEG displayed a sharp contrast between strong and weak activity, whereas MEG‐estimated SEEG exhibited a smoother distribution.

Resting‐state functional connectivity in the alpha band followed a similar trend as in P1 and P9. *Seed‐based connectivity*: Spatial correlations across all seeds were: *AEC*(0.42±0.18), *OAEC*(0.24±0.24), *PLV*(0.31±0.18), and wPLI*(‐0.01±0.11) (Figure 4I–L). *Whole connectome* (Figure 4M–P): Significant spatial correlations were found for *AEC*(0.39), *OAEC*(0.33), and *PLV*(0.28), while wPLI* showed no significant correlation (‐0.01).

For the other six patients, detailed results are presented in Figures S1–S6.

### 2.5 Summary Statistics for Spike Localization and the Effect of Source Depth

**FIGURE 5:**
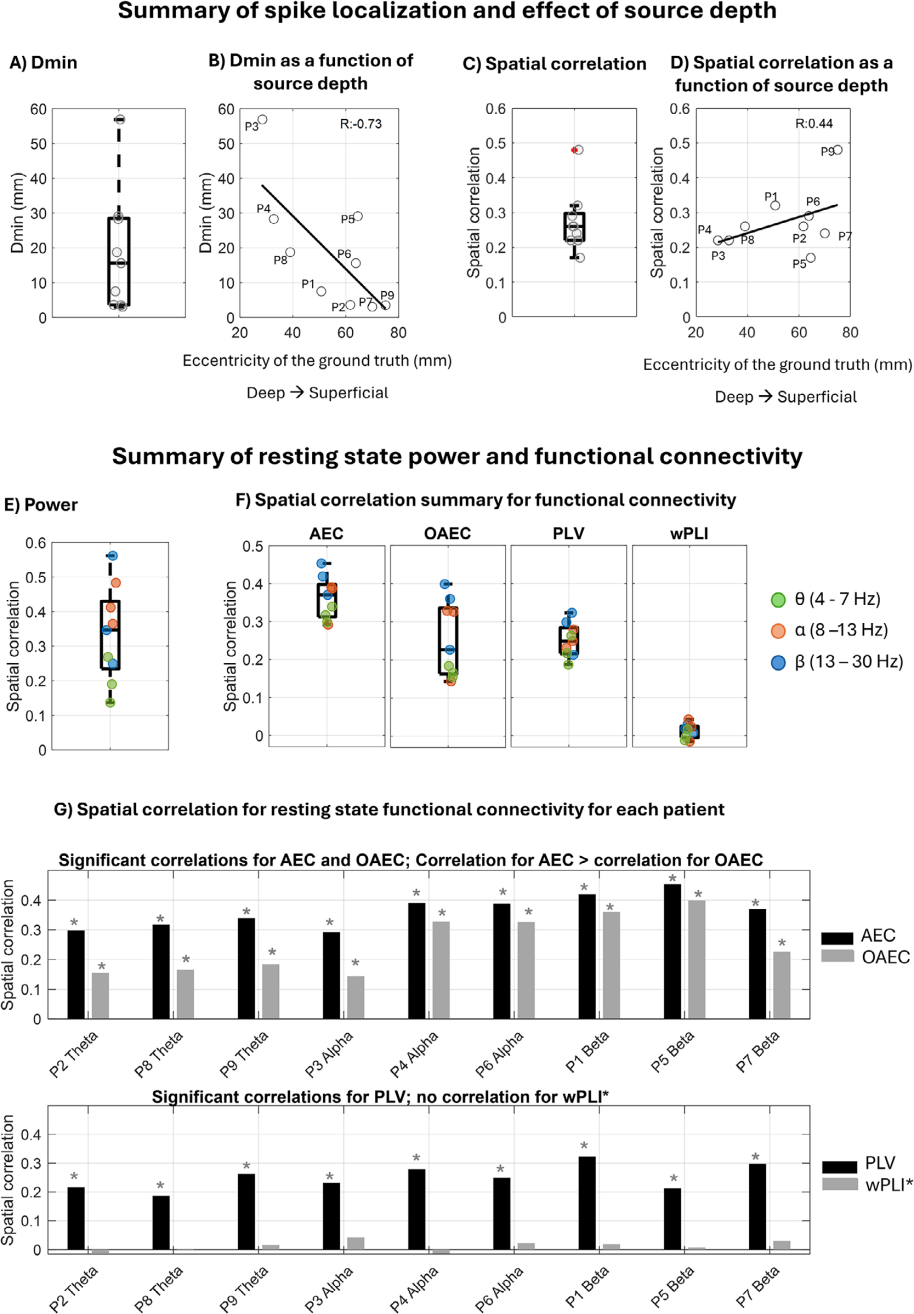
Summary of localization error (Dmin) and spatial correlation between SEEG and MEG‐estimated SEEG for spike and resting‐state results in nine patients. Spike localization (A–D): (A) Dmin values summarized as a boxplot distribution. (B) Dmin as a function of source depth, expressed by the eccentricity of the SEEG channel with maximum spike activity. Low eccentricity values correspond to deep sources, and high eccentricity values to superficial sources (Pearson correlation = −0.73, *p* < 0.05). (C) Boxplot of spatial correlations between MEG‐estimated SEEG and SEEG for spike localization. (D) Spatial correlations between MEG‐estimated SEEG and SEEG at the spike peak as a function of source depth (Pearson correlation = 0.44, *p* = 0.23). Resting state analysis (E–G): (E) Spatial correlations between SEEG and MEG‐estimated SEEG for resting‐state power. Frequency bands are color‐coded: green for theta, red for alpha, and blue for beta. (F) Spatial correlations for functional connectivity metrics (AEC, OAEC, PLV, and wPLI*) when considering the whole connectome. (G) Spatial correlations for resting‐state connectivity per patient, shown as a bar plot. Spatial correlations that exceeded the 95% range of the null distribution (i.e., statistically significant) are marked with a (^★^).

Across the nine patients, assessing spike localization at the averaged spike‐peak, the median *Dmin* between MEG‐estimated SEEG and ground truth SEEG (averaged SEEG spike‐peak) was 15.6 ± 12.1 mm (median±median absolute deviation (MAD)) (Figure 5A). *Dmin* was less than 30 mm for 8 of 9 patients, and exhibited a statistically significant negative correlation with source depth (Pearson correlation = −0.73, *p*<0.05) (Figure 5B). Source depth was quantified using the eccentricity of each SEEG contact, the distance from the contact to the center of the head. As expected, deeper sources tended to have higher localization errors. The spatial correlation between SEEG and MEG‐estimated SEEG exhibited a comparable depth‐dependent trend, though the effect was not statistically significant (Pearson correlation = 0.44, *p* = 0.23) (Figure 5C, D). For nine patients, the *AUC* assessing the spatial accuracy of spike localization at the averaged spike‐peak was 0.67 ± 0.06 (median±MAD) (Figure S7). No depth dependency was observed for AUC (Pearson correlation = 0.08, *p* = 0.8). Analysis of single‐spike localization is reported in the Section S1 and Figure S8. In some patients, large localization errors were observed because the selected MEG spikes were not clearly visible in SEEG.

### 2.6 Summary Statistics for Resting‐State Analysis

The median spatial correlation between SEEG and MEG‐estimated SEEG for resting‐state oscillatory power was 0.35 ± 0.14 (Figure 5E). A trend toward higher correlations was observed for alpha and beta compared to the theta band. All patients showed statistically significant correlations between SEEG and MEG‐estimated SEEG, except for P2 in the theta band, for whom most of the power was localized in posterior regions, whereas the implantation mainly covered frontal regions (Figure S1).

Figure 5F displays the spatial correlations obtained using whole connectome comparisons, for functional connectivity computed using *AEC*, *OAEC*, *PLV*, and wPLI*. For amplitude‐based metrics, OAEC showed lower spatial correlation than *AEC* (*AEC*:0.37 ± 0.07; *OAEC*:0.23 ± 0.12), however, both remained statistically significant. For phase‐based metrics, *PLV* yielded significant correlations (0.25 ± 0.05), but wPLI* correlations were close to zero and not statistically significant (0.02 ± 0.02). For connectivity, all patients were consistent: *OAEC* correlations were lower than *AEC*, but *AEC* and *OAEC* exhibited statistically significant correlations (Figure 5G). *PLV* also yielded significant correlations across all patients, whereas wPLI* did not show significant correlations in any case. When we repeated the analysis using the original definition of *wPLI* [41], no significant correlations were observed for any patient.

So far, we have presented the spatial correlations between SEEG and MEG‐estimated SEEG connectivity across different metrics. To further evaluate the performance of these measures, we examined the raw connectivity values in detail in the Section S2 and Figure S9.

## 3 Discussion

We present a comprehensive validation of MEG source imaging for localizing transient epileptic spikes and low signal‐to‐noise ratio (SNR) resting‐state background activity, including ongoing oscillations and functional connectivity patterns. We analyzed a unique dataset of simultaneous MEG and SEEG [28], considering SEEG as the ground truth for quantitative validation. By employing the MEM method to solve the inverse problem and projecting MEG source maps from the cortical surface to SEEG space, our pipeline enables direct comparisons between MEG‐derived virtual SEEG and actual SEEG at each electrode contact. We found that MEG source imaging using the cMEM method successfully reconstructed averaged epileptic spikes with a median localization error of ∼15 mm and an *AUC* of ∼0.67 for the localization of the local maximum, although the localization of deep generators showed larger errors. For resting‐state activity, relative power in the alpha and beta was estimated more accurately than in slower rhythms such as theta. MEG consistently showed higher connectivity than SEEG for amplitude and phase‐based metrics sensitive to zero‐lag connections (*AEC*/*PLV*), suggesting stronger source leakage [21]. In contrast, connectivity values were found in a more similar range when considering leakage‐corrected metrics (*OAEC*/wPLI*). Among the leakage‐corrected metrics, connectomes estimated using amplitude‐based metric *OAEC* aligned relatively well with SEEG, whereas those derived from phase‐based metrics (wPLI*) were inaccurate. These findings are largely consistent with our previous group‐level results using non‐simultaneous MEG and the MNI iEEG atlas [24, 25].

### 3.1 Localization of Interictal Epileptic Discharges

The localization errors for the nine patients with epileptic spikes were 15.6 ± 12.1 mm (median±MAD) (see Section S1 for single spikes analysis). This level of accuracy is consistent with previous MEG studies and ground truth obtained from non‐simultaneous intracerebral EEG [37, 42, 43] and studies employing high‐density EEG and simultaneous stereo‐EEG electrical stimulations [44, 45]. In Abdallah et al. [37], we compared EEG‐MEG source imaging of epileptic spikes from 17 patients with focal epilepsy against non‐simultaneous SEEG, using a methodology similar to the present study for converting EEG‐MEG sources into virtual SEEG. The reported localization error was 20.7 ± 15.2 mm (mean±standard deviation). Unlike that study, here we applied a depth‐weighted version of the source imaging method [11] and improved MEG localization accuracy.

Tamilia et al. [43] evaluated MEG source imaging using non‐simultaneous iEEG in 24 children with epilepsy, reporting localization errors of 15.4 ± 12.2 mm using Equivalent Current Dipoles (ECD) and 20.7 ± 13.1 mm using Dynamic Statistical Parametric Mapping (dSPM) [46]. In that study, the ground truth was derived from iEEG source imaging, and individual spike maps were combined into a single dSPM map, whereas we validated MEG sources against simultaneously recorded SEEG and localized averaged spikes per patient (single‐spike results are reported in Figure S8).

A key difference in our study is that spikes were selected based on MEG, and the corresponding SEEG were extracted regardless of whether a clear spike was visible in SEEG. As a result, the averaged SEEG did not consistently show sharp peaks. In some cases, the SEEG average peaked earlier or was absent altogether, unlike in our previous non‐simultaneous study [37], where high‐SNR spikes were selected in both modalities. This reflects a deliberate design choice: we aimed to validate MEG while remaining blind to SEEG, whereas other simultaneous studies focused their interest on SEEG discharges and their visibility at the scalp level [26, 28, 31]. Restricting analysis to spikes visible in both modalities would have reduced localization errors (see patient P3 in the Supporting Information), but this was not our objective.

Further evidence on source localization accuracy comes from studies using intracerebral stimulation of a focal dipolar source as ground truth [44, 45], which reported localization errors ranging from a few millimeters to several centimeters. However, such stimulations produce focal, high‐SNR sources, while real epileptic activity is spatially extended, noisier, especially for deep generators [45, 47], and can also propagate to unsampled regions.

In epilepsy, recovering the spatial extent of the generator is of great importance. The MEM technique can recover generators together with their extent [11, 14, 37]. In our current study, the AUC across 9 patients was 0.67 ± 0.06 (median±MAD), consistent with Abdallah et al. [37], who reported AUC:0.71 ± 0.12 (mean±SD) using non‐simultaneously acquired MEG and SEEG and the selection of clear high SNR spikes. Unlike Abdallah et al. [37], where ground truth was visually defined from SEEG spikes, here, spikes were marked in MEG, and SEEG ground‐truth channels were defined using a 30% amplitude threshold from averaged SEEG spikes (∼24 ± 6 channels per patient). AUC values around 0.67 to ≥0.74 (Figures 2, 3, 4 and Sections S1–S6) corresponded visually to good‐to‐excellent spatial overlap between MEG and SEEG maps.

### 3.2 Deep Source Localization

The ability of EEG and MEG to detect deep brain activity, particularly in MEG, has long been debated [3]. However, recent studies have provided compelling evidence that activity from deep brain structures can be recorded using EEG [26, 32] and MEG [8, 28, 48]. While deep sources may generate signals detectable at the scalp, these typically have low SNR and are often masked by stronger superficial activity, becoming largely invisible. Consequently, detecting and localizing such signals remains challenging for any source imaging method. MEG and EEG sensors are inherently more sensitive to superficial than deep generators, leading to systematic underestimation of deep source contributions. To mitigate this, depth‐weighting strategies have been incorporated into source imaging methods to enhance sensitivity to deep structures [49].

We used a depth‐weighted version of MEM, a method we recently proposed and validated to improve deep source localization in EEG and MEG [11]. In that work, we showed that adding depth weighting and incorporating hippocampal meshes into the source space significantly improved localization accuracy for deep sources. Nevertheless, we also observed persistent localization bias in the form of spurious superficial activity. This suggests that even for high‐amplitude spikes, it remains difficult to determine whether generators are exclusively deep or involve both deep and superficial structures. Advanced signal processing techniques to separate deep from superficial generators could be considered for this purpose [8, 28, 50].

### 3.3 Resting‐State Power

Significant spatial correlations between SEEG and MEG‐estimated power were found for all patients except P2 in the theta band. For P2, most of the MEG theta power was localized in posterior regions, whereas the SEEG implantation mainly covered frontal regions. Qualitatively, distributions of theta, alpha, and beta power resembled previously reported maps [22, 23, 24]: theta was broadly distributed as in iEEG at the group level [24], alpha was concentrated over posterior regions, and beta was strongest over the pre‐ and post‐central regions [24, 51]. These results reflected our group‐level findings [24], which compared MEG and iEEG using non‐simultaneous data: resting‐state MEG from a healthy cohort was validated against an iEEG atlas [22]. In Afnan et al. [24], relative power in each iEEG region was highly distinct, showing strong contrasts across regions, whereas MEG‐estimated maps were smoother. This smoothing was also evident in our current study, particularly in the alpha and theta bands (Figures 3 and 4).

In Afnan et al. [24], MEG from healthy subjects showed the dominant peak around 10–12 Hz (consistent with Mahjoory et al. [52]), whereas the iEEG peak was around 7–8 Hz in epilepsy patients (only considering normal contacts). Similarly, Groppe et al. [53] reported that intracranial electrocorticography from 15 epilepsy patients showed a dominant resting‐state theta (∼7 Hz), rather than the ∼10 Hz alpha typically observed in EEG/MEG. This discrepancy may reflect slower alpha in patients compared to healthy subjects [54]. Here, although we did not systematically analyze peak frequencies, MEG and SEEG showed similar oscillatory peaks within each patient, suggesting that the alpha differences in Afnan et al. [24] primarily arose because MEG was recorded in healthy subjects, while iEEG, even from selected “healthy” regions, came from epilepsy patients. Formal validation of these observations remains a topic for future studies.

### 3.4 Compromise Between Removing Spurious Connectivity and Genuine Zero‐Lag Connectivity

Due to the issue of source leakage inherent in EEG/MEG‐derived connectivity estimates, it is often recommended to use metrics that eliminate zero‐lag connections to obtain more interpretable results [4, 9, 40]. However, this approach may discard some genuine zero‐lag interactions. Several studies have investigated the reliability of connectivity metrics, either by assessing test‐retest reproducibility [55, 56] or by comparing EEG/MEG‐derived connectomes with fMRI‐derived connectomes [18, 19]. Colclough et al. [56] found orthogonalized *AEC* and partial correlation to be the most consistent corrected metrics, while phase‐based measures performed poorly. Garcés, et al. [55] similarly reported greater reliability for *AEC* and *PLV* than for leakage‐corrected metrics. Similar findings were obtained with source‐localized EEG [57]. These studies suggest that uncorrected metrics may overestimate reliability due to leakage. Leakage‐corrected metrics are generally recommended despite their lower reproducibility [4]. These studies also highlight the complexity of assessing connectivity metrics in EEG/MEG research and emphasize the importance of careful interpretation of results, especially in the context of resting‐state data.

Unlike approaches that rely on reproducibility or test‐retest reliability across subjects and sessions [9, 55, 56] or comparisons with fMRI [18, 19], which reflect different brain mechanisms, we previously validated MEG‐derived connectivity in healthy participants using an iEEG atlas [25]. In that group‐level study, MEG exhibited higher connectivity than iEEG for *AEC* and *PLV*, suggesting an overestimation due to leakage. The spatial correlation between MEG and iEEG connectomes was moderate to low. For leakage‐corrected metrics (*OAEC* and wPLI*), overall connectivity magnitudes were more comparable, but spatial correlations decreased. Correlations remained significant for *OAEC* in the alpha, beta, and high‐gamma bands, and for wPLI*, across all bands. *OAEC* also remained significant for deep contacts in alpha and beta, whereas wPLI*, was not accurate. A key limitation of that study was the lack of simultaneous data: MEG was recorded in healthy participants, while iEEG was obtained from patients with epilepsy, constructed from channels in regions considered healthy. This limitation was unavoidable, as iEEG data are not available from healthy individuals.

Our current results from simultaneous MEG and SEEG largely replicated our previous findings [25], except for wPLI*. The connectivity values were comparable to those reported previously [25] (see Section S2 and Figure S9). MEG connectivity derived from *AEC* and *PLV* was higher than that from SEEG, reflecting the influence of source leakage. For leakage‐corrected metrics (*OAEC* and wPLI*), connectivity magnitudes were more similar across modalities (Figure S9), though spatial correlations were lower than for uncorrected metrics (*AEC*,  *PLV*). Among the corrected metrics, *OAEC* correlations remained significant in all patients, showing that meaningful connectivity can be recovered with MEG. In contrast, wPLI* results differed from our group‐level study; here, correlation was near zero or even negative, suggesting inaccurate MEG estimates. This pattern persisted when we repeated the analysis using the original *wPLI* definition [41], which includes envelope amplitudes (results not shown).

Although several studies have reported poor test‐retest reliability for leakage‐corrected phase‐based metrics [55, 56], others have demonstrated that *wPLI* can effectively reveal connectivity. *wPLI* has been applied in task‐based paradigms [58], in characterizing brain networks upon awakening from slow‐wave sleep [59], and during seizures [60]. However, because these data were task‐evoked, the signals were much stronger than during the resting state. *wPLI* may therefore be more robust for estimating connectivity in evoked or task‐based studies than in resting‐state analyses at a whole‐brain connectome level.

Further insight comes from iEEG connectome studies. Williams et al. [61] showed that iEEG connectomes computed with *PLV* revealed anatomically contiguous modules, unlike the distributed patterns observed in resting‐state fMRI, suggesting that *PLV*‐based iEEG connectivity is largely local. This may explain why MEG‐derived connectomes using *wPLI* were inaccurate: if phase‐based metrics mainly capture local connections, removing zero‐lag components can produce noisy connectomes that exclude genuine synchrony. In contrast, amplitude envelope‐based metrics, which reflect large‐scale synchronization, capture long‐range connections and recover distributed networks similar to resting‐state fMRI [62]. In our study, leakage‐corrected amplitude envelope‐based metrics (*OAEC*) estimated MEG connectomes relatively more accurately, consistent with Colclough et al. [56].

### 3.5 Moderate to Low Correlations Between MEG and SEEG Connectome

The correlations between MEG‐ and SEEG‐derived connectomes were moderate to low for *AEC*, *OAEC*, and *PLV*, ranging from 0.14 to 0.45. In our previous group‐level study using non‐simultaneous data from healthy participants and an iEEG atlas [25], spatial correlations between MEG‐estimated and atlas‐derived iEEG connectomes also ranged from 0.15 to 0.38 across theta, alpha, and beta bands. While one might expect higher correlations in the current simultaneous MEG‐SEEG study, differences between the datasets must be considered. In the earlier study, MEG and iEEG came from different populations, and the iEEG connectome was constructed across subjects using 76 region‐of‐interest (ROIs), with a variable number of channels per ROI. Despite these limitations, cross‐modal correlations suggested that group‐level ROI comparisons can yield meaningful connectivity estimates. Previous work, including simultaneous EEG‐fMRI recordings [19], indicated that averaging across subjects enhances cross‐modal correlations by reducing subject‐specific noise, a pattern also observed in our prior atlas‐based MEG/iEEG study [25]. Consequently, even with simultaneous recordings at the channel level, correlations may remain modest, highlighting the influence of noise at a single‐subject level. The lack of an atlas‐based framework for simultaneous MEG‐SEEG currently limits ROI‐level analysis at the population level, but if such a dataset becomes available, this hypothesis could be tested more directly.

### 3.6 Clinical Relevance

Our overall findings have direct clinical relevance for pre‐surgical evaluation in epilepsy, demonstrating that MEG can reliably localize interictal activity and thus complement SEEG in guiding surgical decisions. Our results also highlight an important limitation: MEG may mislocalize activity originating from deep structures, underscoring the need for cautious interpretation of source localization in both clinical and research applications, including studies of disorders where deep regions such as the hippocampus play a critical role. Accurate mapping of resting‐state oscillations and connectivity also opens perspectives for identifying pathological network alterations, with potential utility in both prognosis and surgical planning [23]. Finally, the simultaneous MEG‐SEEG framework provides a unique opportunity to benchmark non‐invasive measures against intracranial recordings, thereby enhancing their interpretability across both clinical and basic research contexts. This framework can also be extended to the validation of other electrophysiological biomarkers obtained using non‐invasive EEG/MEG, such as high‐frequency oscillations or fast oscillations concomitant with spikes, which may further improve the characterization of epileptogenic networks [63, 64, 65].

### 3.7 Limitations and Perspectives

The limited number of patients reduces the generalizability of our findings. Comparisons across frequency bands were not feasible, as we focused exclusively on the dominant frequency band in each patient. Additionally, our analysis was restricted to 1 min of resting‐state activity, with power and connectivity measures averaged over this period. While the use of simultaneous MEG and SEEG offers a unique opportunity to examine the dynamics of brain activity across modalities, such analyses were beyond the scope of the present study. The sample size was also insufficient to support region‐of‐interest (ROI) based analyses, and spatial coverage was limited. Increasing the number of participants in future studies will improve cortical and subcortical sampling, enabling more comprehensive assessments of the whole brain.

A limitation of this study is that resting state segment selection was performed visually to ensure artifact‐ and discharge‐free data, which may influence power and connectivity estimates. Although this approach is commonly used in intracranial recordings, future work should assess the reproducibility of the findings across different segment selections.

In this study, we tested the statistical significance of cross‐modal correlations between MEG‐ and SEEG‐derived connectomes using a permutation‐based null model generated by randomly permuting the anatomical labels of MEG‐estimated SEEG channels. However, this approach destroys spatial structure and does not preserve spatial autocorrelation or distance‐dependent effects that may influence connectivity measures. Future studies could incorporate spatial autocorrelation‐preserving null models, such as spatial permutation (‘spin’) tests [66], or generative null models based on Moran spectral randomization [67] to provide a more stringent evaluation. However, these approaches would require adaptation to account for the limited and sparse spatial sampling inherent to SEEG when assessing spatial autocorrelation or distance‐dependent effects.

A methodological choice in our study is that source reconstruction is constrained to the cortical grey matter surface, after which the reconstructed activity is projected onto SEEG contacts using a biophysical SEEG forward model. This choice is motivated by the physiological origin of EEG and MEG signals, which arise primarily from synchronized neuronal populations in cortical grey matter. In contrast, full volumetric source spaces, such as those considered in some beamformer‐based virtual electrode approaches [68, 69], may localize activity to white matter regions, potentially leading to physiologically implausible estimates at contacts located outside grey matter. The use of the SEEG forward model allows cortical generators reconstructed by MEG to contribute primarily to nearby SEEG contacts located close to grey matter, while contacts farther from the cortical surface receive lower projected activity, as signal strength decreases with the inverse of the square of the distance. Our approach also models the relationship between specific orientations of dipolar sources explicitly along the cortical surface and corresponding SEEG contacts (see Section S3). We therefore believe that this approach provides a more physiologically plausible estimation of virtual SEEG activity than a fully volumetric 3D grid approach, where activity may be reconstructed to white matter regions.

We used SEEG as ground truth to validate MEG source imaging, but its limited spatial coverage meant validation was partial at the single‐subject level. Some spikes marked in MEG may have been missed in SEEG due to restricted implantation. Moreover, starting from MEG could mix multiple spike types generated in nearby but different SEEG electrodes, leading to similar MEG topographies that are difficult to distinguish. This mixing may reduce apparent localization accuracy and obscure differences between spike generators, although it reflects real‐life marking in routine clinical practice. In contrast, a selection strategy starting from SEEG and restricting the analysis to spikes clearly visible in both modalities could show a higher localization accuracy. However, this was not the focus of the present study, as we aimed to validate MEG‐derived estimates in a practical setting, when SEEG is not available.

Beyond validation, combining SEEG and MEG for complementary use has strong potential. Multimodal fusion could overcome limited SEEG sampling and detect interictal discharges missed by SEEG alone [70]. Our group previously implemented EEG‐MEG fusion source imaging within the MEM framework [71], and this approach could be extended to integrate SEEG source localization, optimally combining complementary recordings. Moreover, projecting MEG‐reconstructed SEEG activity beyond implanted SEEG contacts to estimate activity in unsampled regions could potentially help identify regions that may benefit from additional SEEG electrode implantation, a topic we plan to investigate in future work.

## 4 Conclusions

By leveraging simultaneously acquired MEG and SEEG recordings in nine patients, we provide a methodological pipeline that enables direct, quantitative comparison of MEG source imaging and connectivity estimates against intracranial benchmarks. MEG source localization of epileptic generators was found to be reasonably accurate, with a median localization error of 15 mm and an AUC of 0.67, although deep sources were associated with larger errors. For resting‐state activity, spatial correlations between MEG and SEEG for relative power estimates were moderate, with a trend toward higher agreement in the beta and alpha bands compared to the theta band. In terms of functional connectivity, MEG systematically overestimated connectivity relative to SEEG when using metrics that retain zero‐lag connectivity (*AEC*/*PLV*), more likely because of the importance of source leakage bias. Among the leakage‐corrected metrics, orthogonalized amplitude envelope correlation (*OAEC*) provided statistically significant cross‐modal MEG‐SEEG correlations, whereas the phase‐based metric wPLI* yielded poor cross‐modal agreement and should be interpreted with caution. These results highlight the strengths and limitations of MEG for non‐invasive mapping of brain activity.

## 5 Materials and Methods

### 5.1 Simultaneous SEEG‐MEG Data

Simultaneous MEG and SEEG were recorded at the Marseille MEG center (Institut de Neurosciences des Systèmes, AMU, Inserm), located within the Epileptology and Cerebral Rhythmology unit. Participants provided written informed consent for the simultaneous MEG/SEEG recording and for the publication of data, and an institutional ethical review committee approved the research (Comité de Protection des Personnes Sud Méditerranée, ID RCB: 2012‐A00644‐39). The technical setup of simultaneous MEG/SEEG recordings was described in Badier et al. [27]. MEG was acquired using a 4D Neuroimaging 3600 whole‐head system (248‐magnetometers) at a sampling rate of 2034.51 Hz. Patients were lying down and instructed to relax with their eyes closed during acquisition. Simultaneous SEEG was recorded on 256 channels (256‐channel BrainAmp, Brain Products GmbH, Munich, Germany), sampled at 2048–2500 Hz. The number of electrodes implanted per patient varied (median±MAD: 208±13), and each SEEG electrode contained 10 to 15 contacts.

Resting‐state data were obtained from a task‐free run (∼10–15 min). Segments corresponding to specific frequency bands were visually identified and selected based on the absence of artifacts and epileptic discharges in the MEG signal. Segment lengths were variable and subsequently concatenated, and the first 60 s of the resulting signal were retained for analysis. On average, 10 ± 5.4 segments per patient were selected, yielding approximately 60–80 s of usable data per subject.

### 5.2 SEEG Contact Localization

Each patient underwent a pre‐operative T1‐weighted MRI on a 3T MRI system and a post‐implantation CT scan. The two scans were co‐registered using GARDEL software (https://meg.univ‐amu.fr/doku.php?id=epitools:gardel) [28, 72] to precisely localize the SEEG contacts within each patient's MRI space. Brain segmentation and reconstruction of the white/gray matter interface were obtained using *recon‐all* from FreeSurfer software [73].

### 5.3 MEG Source Space and Forward Model Estimation

Coregistration of MEG sensors with anatomical MRI and construction of the source and forward models were performed in Brainstorm [74] using cortical and subcortical surfaces reconstructed using FreeSurfer. The source space included the cortical middle‐layer mesh (between white/gray matter interface and gray matter/pial surface interface) and both hippocampi, with dipole sources placed along their surfaces and oriented orthogonally at each point; the combined cortical and hippocampal surfaces were then downsampled to ∼8000 vertices. The forward model was computed using OpenMEEG software [75] implemented in Brainstorm using a 3‐layer Boundary Element Model (BEM) [76] consisting of brain, skull, and scalp surfaces with conductivity values of 0.33, 0.0165, and 0.33 S m^−1^ [77].

### 5.4 MEG and SEEG Data Preprocessing

The preprocessing included applying an online correction based on reference channels for MEG, a bandpass filter between 2 and 80 Hz, a notch filter at 50 Hz, noisy channels removal, and downsampling data to 600 Hz for spikes analysis and 200 Hz for resting‐state analysis. For MEG‐SEEG realignment, regular triggers with time jitters (inter‐trigger range 3000–3500 ms) were sent to both SEEG and MEG systems. Signals were co‐registered offline based on these triggers, with in‐house code written in Matlab.

### 5.5 MEG Source Imaging Using Maximum Entropy on the Mean (MEM)

The MEG inverse problem was solved using the Maximum Entropy on the Mean (MEM) [14]. MEM provides accurate localization of the generators together with their spatial extent, as demonstrated by the standard variant of MEM, cMEM [14, 37], as well as the wavelet‐based extension, wMEM [24, 35]. For coherent MEM (cMEM), “coherent” refers to the use of a coherent spatial prior—a data‐driven parcellation that remains fixed over time [14, 36, 37]. wMEM is specifically designed to localize brain oscillatory patterns [24, 35] by applying a discrete wavelet transformation (Daubechies wavelets) to characterize the oscillatory patterns in the data before applying the MEM solver [35].

For localizing spikes, we applied cMEM, using a depth weighting parameter we proposed and validated for accurate localization of deep brain activity [11]. For resting‐state activity, we used a wMEM version adapted for localizing low‐SNR background activity [24], whose accuracy was validated at the population level and considering a broad volumetric coverage using the MNI iEEG atlas [24, 25] for MEG, as well as for high‐density EEG recordings [78]. For both cMEM and wMEM, the depth weighting parameter was set to 0.5.

For spike localization, a 2 s artifact‐free baseline was used to compute the noise covariance. In contrast, because resting‐state data lack a clear baseline, we estimated the noise covariance by generating a quasi‐synthetic baseline: the signal was phase‐randomized in the Fourier domain [79] and processed using a 1 s sliding window, allowing precise estimation of noise covariance for each wavelet sample across time scales (see Afnan et al. [24] for details). cMEM and wMEM implementations are available in Brainstorm [74] (https://neuroimage.usc.edu/brainstorm/Tutorials/TutBEst/).

### 5.6 Estimation of Virtual SEEG Data From the MEG Source Map

For quantitative comparison of MEG source maps with SEEG, specifically to account for the different spatial sampling and physical units of the two modalities, MEG source maps in nanoAmpere‐meters and SEEG in microVolts, we estimated virtual SEEG electric potentials at each SEEG channel position of a patient that would correspond to MEG source maps, by applying a patient‐specific SEEG forward model to the source maps [36, 37] (details in Section S3).

### 5.7 Frequency‐Specific Relative Power

For each SEEG and MEG‐estimated SEEG channel, the power spectral density (PSD) was estimated using Welch's method (time duration: 0–60 s, 2 s sliding Hamming windows, overlap: 50%). For each channel, the relative PSD was obtained by dividing the power at each frequency bin by the total power across the whole frequency range (2–80 Hz). We averaged the relative power across all frequency bins within the dominant frequency band identified for each patient.

### 5.8 Connectivity Metrics

For SEEG and MEG‐estimated SEEG, connectivity between each channel pair was computed using amplitude envelope correlation (*AEC*) [10], phase locking value (*PLV*) [38, 39], orthogonalized AEC (*OAEC*) [40] and a modified version of the weighted phase lag index (*wPLI*) [41] considering only the phase information, as we previously evaluated in [25] (details in Section S4).

### 5.9 Comparison Between SEEG and MEG‐Estimated SEEG

For spike analysis, we compared MEG‐estimated SEEG and SEEG at the spike‐peak using three metrics: (i) the Euclidean distance between the SEEG channel exhibiting maximum amplitude and the MEG‐estimated SEEG channel with maximum amplitude (*Dmin*); (ii) spatial correlation between MEG‐estimated SEEG and SEEG, and (iii) the spatial overlap as assessed using the area under the ROC curve (*AUC*) [36, 37]. The AUC was computed after thresholding the SEEG map at 30% (to define the ground truth) and varying the amplitude threshold of the MEG‐estimated SEEG to assess spatial overlap.

For resting‐state analysis, we computed Pearson spatial correlations between the SEEG and MEG‐estimated SEEG for the average power map and for connectomes derived from the four connectivity metrics in the respective dominant frequency bands. To assess statistical significance, we generated an empirical null distribution of 5000 cross‐modal correlation values by randomly permuting the anatomical labels of MEG‐estimated SEEG channels, thereby disrupting the spatial correlation structure, and computing the Pearson correlation between SEEG and the spatially permuted MEG for each permutation. A cross‐modal (MEG‐SEEG) correlation was considered significant if it was positive and exceeded the 95% range of the null distribution, centered on the median.

## Author Contributions

**Christian G. Bénar**: supervision, conceptualization, investigation, funding acquisition, data curation, resources, software, writing – review and editing. **Christophe Grova**: supervision, conceptualization, investigation, funding acquisition, resources, software, writing – review and editing, data curation. **Fabrice Bartolomei**: investigation, data curation, resources, writing, review, and editing. **Francesca Bonini**: investigation, writing – review and editing, validation, resources, methodology. **Jean‐Marc Lina**: investigation, methodology, writing, review, and editing. **Jean‐Michel Badier**: investigation, writing, review and editing, methodology, software, resources. **Jean Gotman**: conceptualization, investigation, funding acquisition, writing – review and editing, supervision. **Jawata Afnan**: conceptualization, investigation, writing – original draft, methodology, validation, visualization, formal analysis, software. **Maria Fratello**: conceptualization, investigation, methodology, validation, visualization, formal analysis, writing – original draft, software. **Samuel Medina Villalon**: investigation, writing – review and editing, software, methodology, validation, resources. **Zhengchen Cai**: investigation, validation, writing – review and editing, methodology.

## Conflicts of Interest

The authors declare no conflict of interest.

## Supporting information




**Supporting File**: advs76365‐sup‐0001‐SuppMat.docx

## Data Availability

The data that support the findings of this study are available on request from the corresponding author. The data are not publicly available due to privacy or ethical restrictions.
