# Supplementary material for: Assessing Strengths and Limitations of Magnetoencephalography Source Imaging With Intracerebral EEG
Source: Adv Sci (Weinh). 2026 Jul 8:e76365. Online ahead of print. doi: 10.1002/advs.76365 (PMC13344062; doi:10.1002/advs.76365)
Supplement: Supplementary file 1 — Supporting File: advs76365‐sup‐0001‐SuppMat.docx [file ADVS-9999-e76365-s001.docx]

**Supporting Information for**

Assessing strengths and limitations of magnetoencephalography source imaging with intracerebral EEG.

Jawata Afnan^1,2,3^; Maria Fratello^4^; Francesca Bonini^4,5^; Samuel Medina Villalon^4,5^; Zhengchen Cai^3^; Jean-Marc Lina^6,7^; Jean-Michel Badier^4^; Fabrice Bartolomei^4,5^; Jean Gotman^3^; Christian G. Bénar^4^; Christophe Grova^1,3,6,8^

Correspondence to: Dr. Christophe Grova

Email: [christophe.grova@concordia.ca](mailto:christophe.grova@concordia.ca)

S1 Single spike analysis

We also computed and summarized the single-spike validation results in Supplementary Fig.S8. The localization error (median±MAD) for all spikes in each patient was as follows: P1: 23.2±11.8 mm (number of spikes: 12), P2: 37.0±8.1 mm (number of spikes: 46), P3: 43.0±33.4 mm (number of spikes: 11), P4: 26.9±4.7 mm (number of spikes: 35), P5: 29.9±7.7 mm (number of spikes: 27), P6: 16.4±13.9 mm (number of spikes: 13), P7: 31.3±14.3 mm (number of spikes: 17), P8: 24.3±18.3 mm (number of spikes: 8), and P9: 8.5±8.0 mm (number of spikes: 24). One possible reason for the higher localization errors in some patients was the limited SEEG coverage. Spikes were first identified in MEG, and the same time points were extracted from SEEG for validation; however, in several cases, the selected MEG spikes occurred in areas not well sampled by SEEG electrodes, leading to reduced detectability in SEEG. These spikes were still included in the analysis. For instance, in patient P3, 11 MEG spikes were analyzed, but only 3 were clearly detectable in SEEG, contributing to the high localization error observed for this patient (56 mm for the averaged spike and 43.0±33.4 mm for single spikes). For the 3 spikes clearly visible in SEEG, the localization errors were 20 mm, 13mm, and 27 mm.

S2 Connectivity measures in P1

We plotted the connectivity values obtained from MEG-estimated SEEG and SEEG for all possible channel pairs in Patient 1 (P1) in the beta band, using the four connectivity metrics. P1 had 162 bipolar channels, resulting in 13,041 possible channel pairs. Each point in Fig.S9A represents the connectivity value for one pair. This figure provides an overall view of the distribution and scale of connectivity values in the MEG-estimated versus SEEG-derived connectomes.

We observed that MEG-estimated connectivity values had a broader distribution than SEEG when using metrics that do not correct for zero lag, specifically, AEC (SEEG: 0.06±0.07 vs. MEG: 0.19±0.15) and PLV (SEEG: 0.09±0.02 vs. MEG: 0.19±0.13). In contrast, when using OAEC and wPLI*, which account for and remove zero-lag contributions, MEG and SEEG connectivity values were overall more comparable (OAEC: SEEG 0.05±0.04, MEG 0.08±0.06; wPLI*: SEEG 0.09±0.02, MEG 0.08±0.02).

In Fig.9B, we show connectivity values as a function of the distance between channel pairs for SEEG and MEG-estimated SEEG in P1 (beta band). For AEC and OAEC, connectivity decreased with increasing distance. However, MEG-estimated AEC values were generally higher than SEEG values, since they were contaminated by zero-lag source leakage. After applying orthogonalization (OAEC), connectivity values from both modalities decreased, with a more pronounced drop in MEG than SEEG. PLV also showed a decreasing trend with distance for both modalities, and MEG values remained higher than SEEG. In contrast, for ${wPLI}^{*}$, while a small but decreasing relationship between connectivity strength and inter-channel distance was observed for SEEG, no such relationship was evident for MEG, suggesting in both cases weakly reliable wPLI* measures with resting-state data.

S3 Estimation of virtual SEEG data from the MEG source map

For each patient, to estimate the virtual SEEG potentials from MEG estimated current density along the cortical surface, $\boldsymbol{j}_{MEM}(t)$, (same formula for cMEM or wMEM) we calculated a patient-specific SEEG forward model, G_SEEG_ that estimates the influence of each dipolar source along the cortical surface on each SEEG channel. To do so, our proposed SEEG forward model G_SEEG_ assumes an infinite volume conductor characterized by a conductivity 𝜎 of 0.25 S.m^-1^  as suggested in Cosandier-Rimélé, et al. ^1^. For each patient, for a total number of SEEG contacts *c*, and *n* number of cortical sources (*n* = 8000 vertices), G_SEEG_ is a *c* x *n* matrix that estimates the electrical potential at each SEEG channel *i* (*i* =*1*, *2* …*c*) corresponding to an equivalent current dipole of unit activity located on the vertex *S_j_* and oriented along $\vec{n}$_j_, normal to the cortical surface, calculated as:

$G_{SEEG}\left( i,j \right)=\frac{\vec{n}_{j}.\vec{u}_{ij}}{4\pi\sigma r_{ij}^{2}}$ (1)

where $\vec{u}_{ij}$ is a unit vector oriented from the source *S_j_* to the SEEG channel $i$ and $r_{ij}$ is the Euclidean distance between *S_j_* and contact *i*. To avoid numerical instabilities, when the sources on the cortical surface were too close to the SEEG contacts ($r_{ij}$ < 3 mm), the distance $r_{ij}$ was set to 3 mm instead, keeping the orientation^2^. Finally, we applied this SEEG forward model, G_SEEG_ to the MEG reconstructed source map ($\boldsymbol{j}_{MEM}(t)$) to estimate MEG estimated virtual SEEG potentials on each SEEG channel, MEG estimated SEEG as:

$MEG estimated SEEG(t)= G_{SEEG}\boldsymbol{j}_{MEM}(t)$ (2)

The same conversion was applied to either cMEM results for spike localization or wMEM results for resting-state analysis.

**S4 Connectivity metrics**

Let us consider two signals $X$ and $Y$. To obtain their corresponding amplitude envelope and instantaneous phases, we computed the Hilbert transform for the entire 60-second signals. The Hilbert transform was initially calculated for each 0.5 Hz frequency band and then averaged to obtain one transform for the dominant frequency band of interest for each patient.

$X_{BP,H}$ and $Y_{BP,H}$ ($BP$ stands for bandpass and $H$ stands for Hilbert) are the Hilbert analytical signals of each narrow frequency band for signals $X$ and $Y$, described as $X_{BP,H}\left( t \right)=\left| X_{BP,H}\left( t \right) \right|e^{j\varphi_{X}\left( t \right)}=A_{X}(t)e^{j\varphi_{X}(t)}$ and $Y_{BP,H}\left( t \right)=\left| Y_{BP,H}\left( t \right) \right|e^{j\varphi_{Y}\left( t \right)}=A_{Y}(t)e^{j\varphi_{Y}(t)}$ respectively. Here, $A_{X}(t)$ and $A_{Y}(t)$ denote the instantaneous amplitude of $X_{BP,H}\left( t \right)$ and $Y_{BP,H}(t)$, $\varphi_{X}(t)$ and $\varphi_{Y}(t)$ denote the instantaneous phase of $X_{BP,H}\left( t \right)$ and $Y_{BP,H}(t)$, respectively. We considered the whole 60-second dataset to estimate AEC and OAEC. For PLV and wPLI, we used 6-second epochs and averaged the connectivity over the epochs.

**Amplitude Envelope Correlation (AEC):** AEC between two signals, $X$ and $Y$, is obtained by computing the Pearson correlation between the envelopes of $X_{BP,H}$ and $Y_{BP,H}$. ^3^.

$AEC=\frac{\sum_{t=1}^{T} (A_{X}\left( t \right)-\overline{A}_{X})(A_{Y}\left( t \right)-\overline{A}_{Y})}{\sqrt{\sum_{t=1}^{T} {(A_{X}\left( t \right)-\overline{A}_{X})}^{2}\sum_{t=1}^{T} {(A_{Y}\left( t \right)-\overline{A}_{Y})}^{2}}}$ (3)

Where $T$ is the length of the signal (we considered 60-sec at 200 Hz sampling, $T=12000$ samples) and $\overline{A}_{X}$and $\overline{A}_{Y}$ are the mean values of $A_{X}(t)$ and $A_{Y}(t)$ respectively.

**Orthogonalized Amplitude Envelope Correlation (OAEC):** OAEC was proposed by Hipp, et al. ^4^ following a pairwise orthogonalization between two signals.

$Y_{⏊X}=imag(Y_{BP,H}\frac{{X_{BP,H}}^{*}}{\left| X_{BP,H} \right|})$ (4)

$X_{⏊Y}=imag(X_{BP,H}\frac{{Y_{BP,H}}^{*}}{\left| Y_{BP,H} \right|})$ (5)

Here $*$ means complex conjugate and $imag$ means the imaginary part of the complex number.

We calculated the Pearson correlation between the envelopes of $X_{BP,H}$ and $Y_{⏊X}$. Similarly, the correlation between the envelopes of $Y_{BP,H}$ and $X_{⏊Y}$ is calculated and then the average of these two correlation values is considered as the final OAEC value.

**Phase Locking Value (PLV)**: PLV was originally proposed in Lachaux, et al. ^5^ in the context of evoked activity considering a stable phase-difference along trials. We calculated PLV for each epoch of 6 seconds using an extended definition of PLV (Equation 6), a version proposed by Mormann, et al. ^6^ in the context of resting-state data, by assessing phase locking as a stable phase difference over time:

${PLV}_{X, Y}=\frac{1}{T}\left| \sum_{t=1}^{T} exp(j(\varphi_{X}\left( t \right)-\varphi_{Y}(t))) \right|$ (6)

Where $T$ is the length of the signal (we considered 6-sec epochs at 200 Hz sampling, $T=1200$ samples), $j$ denotes the imaginary unit, $\varphi_{X}\left( t \right)$ and $\varphi_{Y}(t)$ are respectively the corresponding instantaneous phases of signals $X$ and $Y$ at time point $t$. We implemented PLV following the derivation proposed by Bruña, et al. ^7^ as:

${PLV}_{X,Y}=\frac{1}{T}\left| \sum_{t=1}^{T} {\dot{X}_{BP,H}\left( t \right).(\dot{Y}_{BP,H}\left( t \right))}^{*} \right|$ (7)

Where $\dot{X}_{BP,H}\left( t \right)=\frac{X_{BP,H}\left( t \right)}{\left| X_{BP,H}\left( t \right) \right|}$ and $\dot{Y}_{BP,H}\left( t \right)=\frac{Y_{BP,H}\left( t \right)}{\left| Y_{BP,H}\left( t \right) \right|}$.

Finally, we averaged the PLV values across all the epochs.

**Modified Weighted Phase Locking Index (wPLI*):**

The original formula of wPLI was proposed by Vinck, et al. ^8^:

$wPLI=\frac{\left| \sum_{t=1}^{T} A_{X}(t)A_{Y}(t)sin(\varphi_{X}\left( t \right)-\varphi_{Y}\left( t \right)) \right|}{\sum_{t=1}^{T} |A_{X}{\left( t \right)A}_{Y}(t)sin(\varphi_{X}\left( t \right)-\varphi_{Y}\left( t \right))|}$ (8)

If we consider that the amplitudes of the channels in a particular frequency band are stable in time and not involved in the coupling of the channels, the coupling will be strictly described by the phase synchronization and driven by the constant phase shift. Under this assumption, the modified version of wPLI becomes:

${wPLI}^{*}=\frac{\left| \sum_{t=1}^{T} sin(\varphi_{X}\left( t \right)-\varphi_{Y}\left( t \right)) \right|}{\sum_{t=1}^{T} |sin(\varphi_{X}\left( t \right)-\varphi_{Y}(t))|}$ (9)

Where $T$ is the length of the signal (we considered 6s epochs at 200 Hz sampling, $T=1200$ samples). We averaged the ${wPLI}^{*}$ values across all the epochs.

Figures


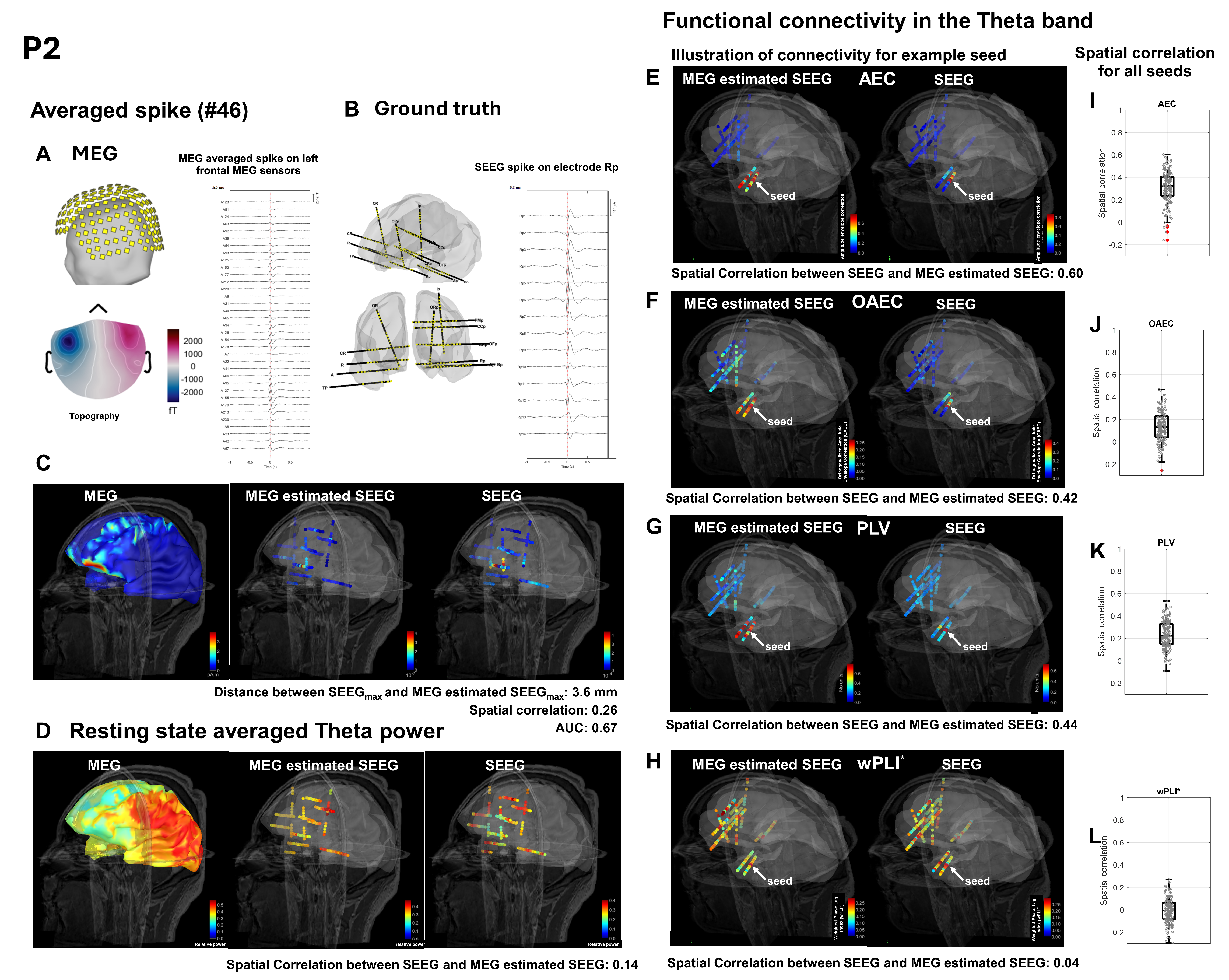


**Fig S1:** Validation of MEG source imaging with simultaneous SEEG for P2. **Spike analysis: (A)** Average of 46 spikes in MEG shown with topography. **(B)** Ground truth: The SEEG implantation and the average of 46 spikes are shown. **(C)** The left panel shows MEG source reconstructed map using cMEM method on the cortical surface at the peak of the spike. The right panel shows the activity of SEEG contacts at the peak of the spike. The middle panel shows the reconstructed source map converted to the SEEG space. The distance between the contact showing maximum activity in SEEG and MEG estimated SEEG is displayed. **Analysis of resting state power in theta band**: **(D)** MEG reconstructed average beta band power is shown on the left panel. The middle panel shows MEG estimated SEEG beta power. The right panel is actual SEEG beta power. The spatial correlation between SEEG and MEG estimated SEEG beta band power is displayed. **Functional connectivity in theta band:** The functional connectivity for an example seed (between the seed and all contacts) is shown for SEEG and MEG estimated SEEG computed using **(E)** Amplitude Envelope Correlation (AEC), **(F)** Orthogonalized Amplitude Envelope Correlation (OAEC), **(G)** Phase Locking Value (PLV) and **(H)** weighted Phase Lag Index (wPLI*). The spatial correlations between SEEG and MEG estimated SEEG for all seeds are summarized as boxplots for **(I)** AEC, **(J)** OAEC, **(K)** PLV and **(L)** wPLI*. Outliers are shown as red dots.


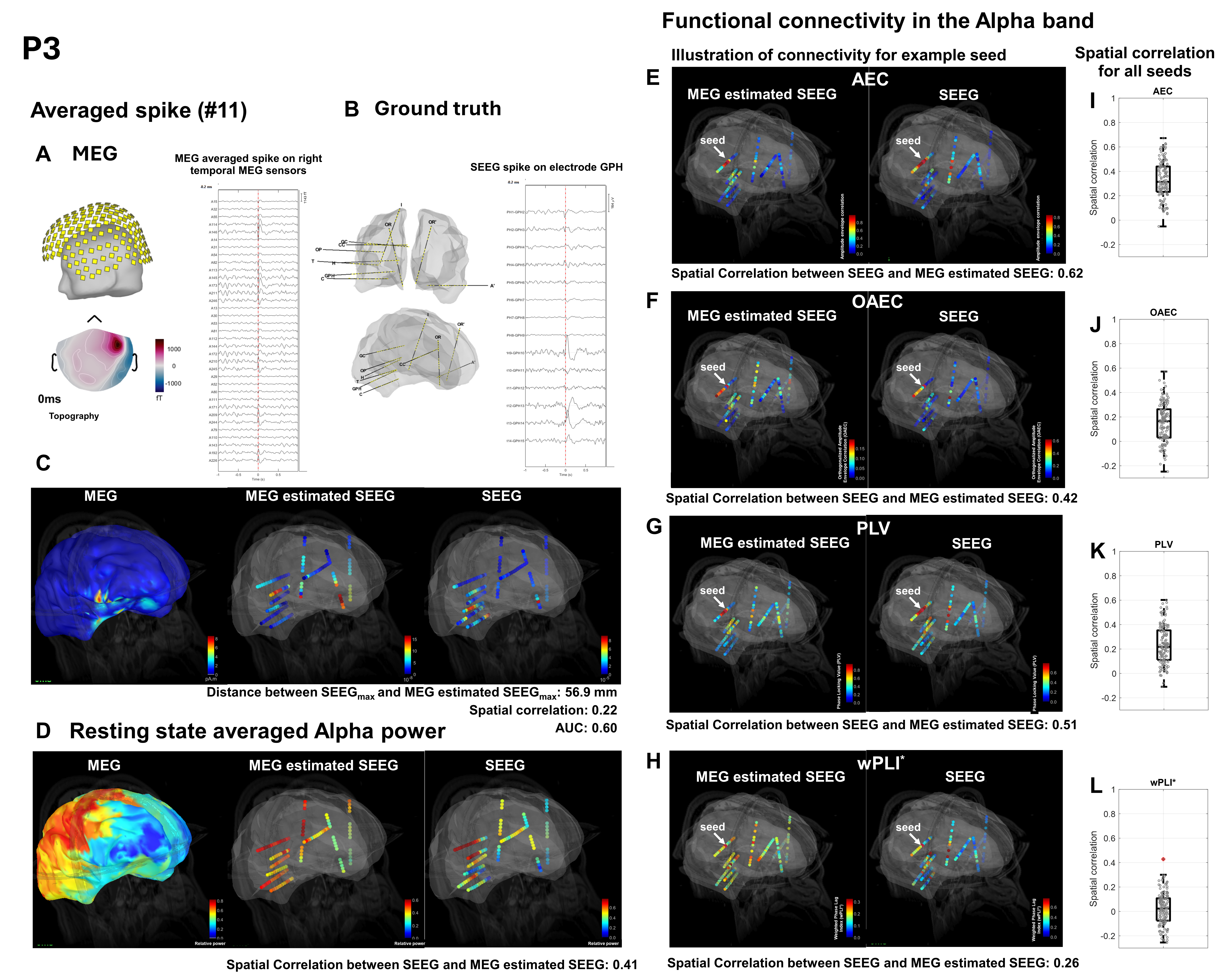


**Fig S2:** Validation of MEG source imaging with simultaneous SEEG for P3. **Spike analysis: (A)** Average of 11 spikes in MEG shown with topography. **(B)** Ground truth: The SEEG implantation and the average of 11 spikes are shown. **(C)** The left panel shows MEG source reconstructed map using cMEM method on the cortical surface at the peak of the spike. The right panel shows the activity of SEEG contacts at the peak of the spike. The middle panel shows the reconstructed source map converted to the SEEG space. The distance between the contact showing maximum activity in SEEG and MEG estimated SEEG is displayed. **Analysis of resting state power in alpha band**: **(D)** MEG reconstructed average beta band power is shown on the left panel. The middle panel shows MEG estimated SEEG beta power. The right panel is actual SEEG beta power. The spatial correlation between SEEG and MEG estimated SEEG beta band power is displayed. **Functional connectivity in alpha band:** The functional connectivity for an example seed (between the seed and all contacts) is shown for SEEG and MEG estimated SEEG computed using **(E)** Amplitude Envelope Correlation (AEC), **(F)** Orthogonalized Amplitude Envelope Correlation (OAEC), **(G)** Phase Locking Value (PLV) and **(H)** weighted Phase Lag Index (wPLI*). The spatial correlations between SEEG and MEG estimated SEEG for all seeds are summarized as boxplots for **(I)** AEC, **(J)** OAEC, **(K)** PLV and **(L)** wPLI*. Outliers are shown as red dots.


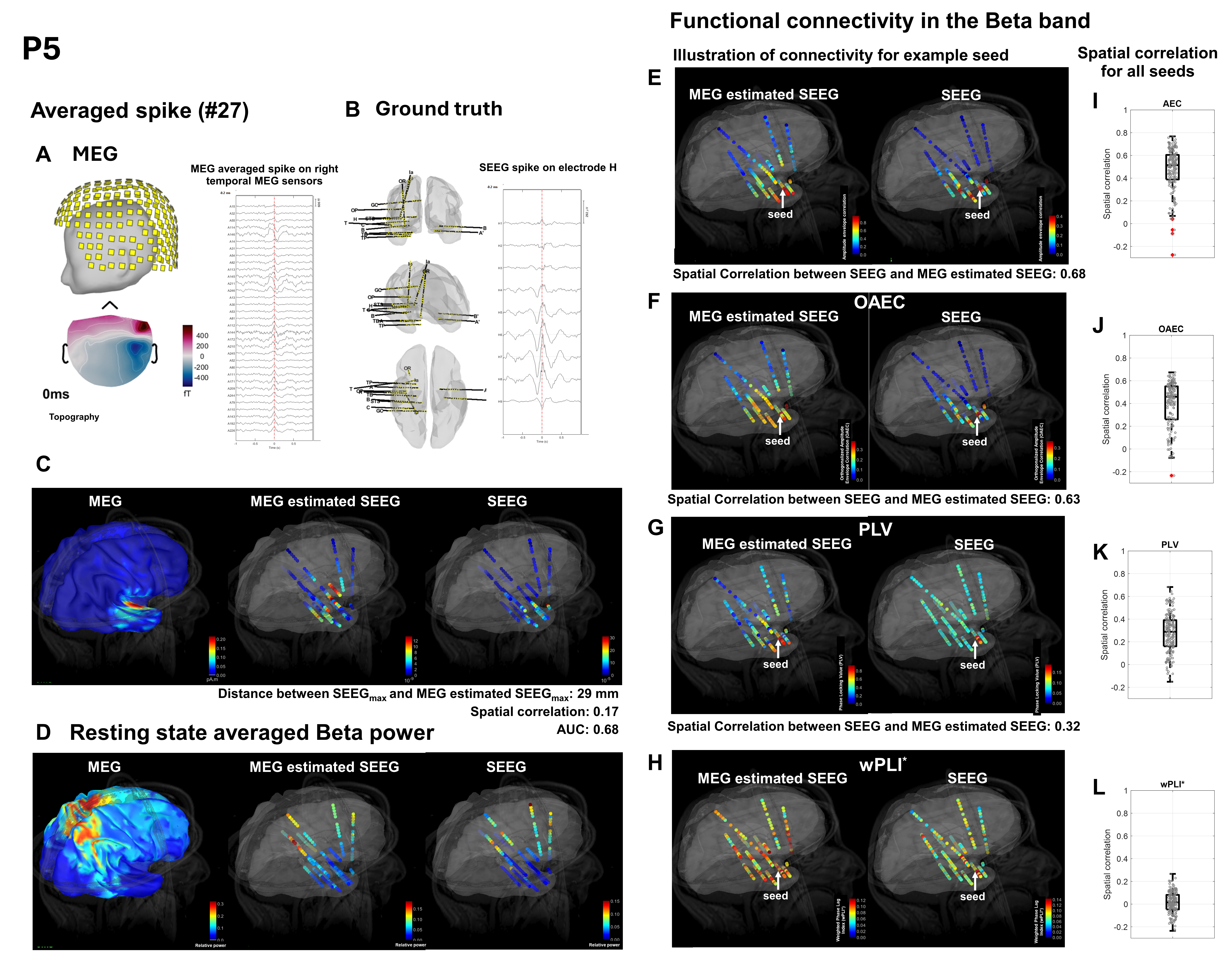


**Fig S3:** Validation of MEG source imaging with simultaneous SEEG for P5. **Spike analysis: (A)** Average of 27 spikes in MEG shown with topography. **(B)** Ground truth: The SEEG implantation and the average of 27 spikes are shown. **(C)** The left panel shows MEG source reconstructed map using cMEM method on the cortical surface at the peak of the spike. The right panel shows the activity of SEEG contacts at the peak of the spike. The middle panel shows the reconstructed source map converted to the SEEG space. The distance between the contact showing maximum activity in SEEG and MEG estimated SEEG is displayed. **Analysis of resting state power in beta band**: **(D)** MEG reconstructed average beta band power is shown on the left panel. The middle panel shows MEG estimated SEEG beta power. The right panel is actual SEEG beta power. The spatial correlation between SEEG and MEG estimated SEEG beta band power is displayed. **Functional connectivity in beta band:** The functional connectivity for an example seed (between the seed and all contacts) is shown for SEEG and MEG estimated SEEG computed using **(E)** Amplitude Envelope Correlation (AEC), **(F)** Orthogonalized Amplitude Envelope Correlation (OAEC), **(G)** Phase Locking Value (PLV) and **(H)** weighted Phase Lag Index (wPLI*). The spatial correlations between SEEG and MEG estimated SEEG for all seeds are summarized as boxplots for **(I)** AEC, **(J)** OAEC, **(K)** PLV and **(L)** wPLI*. Outliers are shown as red dots.


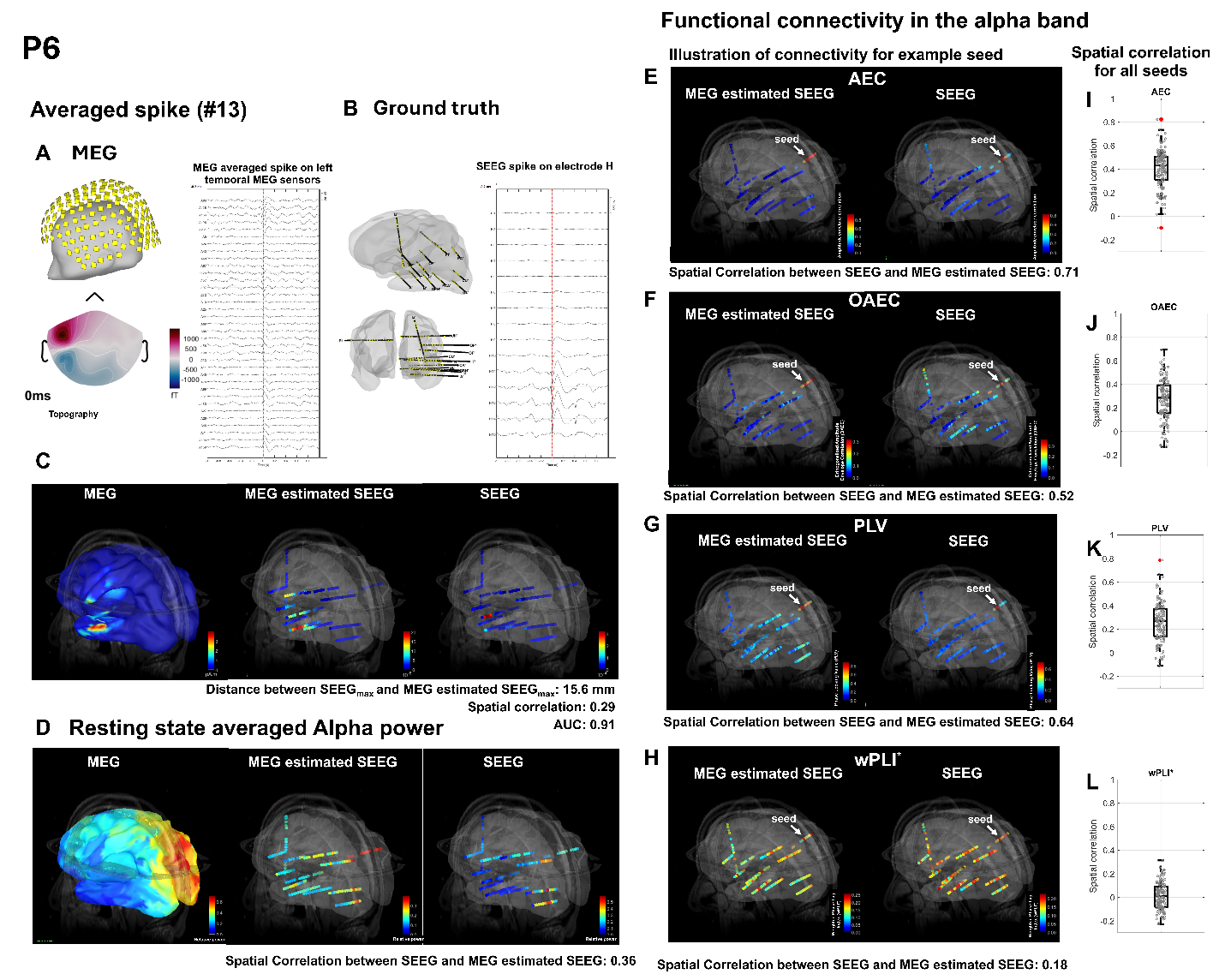


**Fig S4:** Validation of MEG source imaging with simultaneous SEEG for P6. **Spike analysis: (A)** Average of 13 spikes in MEG shown with topography. **(B)** Ground truth: The SEEG implantation and the average of 13 spikes are shown. **(C)** The left panel shows MEG source reconstructed map using cMEM method on the cortical surface at the peak of the spike. The right panel shows the activity of SEEG contacts at the peak of the spike. The middle panel shows the reconstructed source map converted to the SEEG space. The distance between the contact showing maximum activity in SEEG and MEG estimated SEEG is displayed. **Analysis of resting state power in alpha band**: **(D)** MEG reconstructed average beta band power is shown on the left panel. The middle panel shows MEG estimated SEEG beta power. The right panel is actual SEEG beta power. The spatial correlation between SEEG and MEG estimated SEEG beta band power is displayed. **Functional connectivity in alpha band:** The functional connectivity for an example seed (between the seed and all contacts) is shown for SEEG and MEG estimated SEEG computed using **(E)** Amplitude Envelope Correlation (AEC), **(F)** Orthogonalized Amplitude Envelope Correlation (OAEC), **(G)** Phase Locking Value (PLV) and **(H)** weighted Phase Lag Index (wPLI*). The spatial correlations between SEEG and MEG estimated SEEG for all seeds are summarized as boxplots for **(I)** AEC, **(J)** OAEC, **(K)** PLV and **(L)** wPLI*. Outliers are shown as red dots.


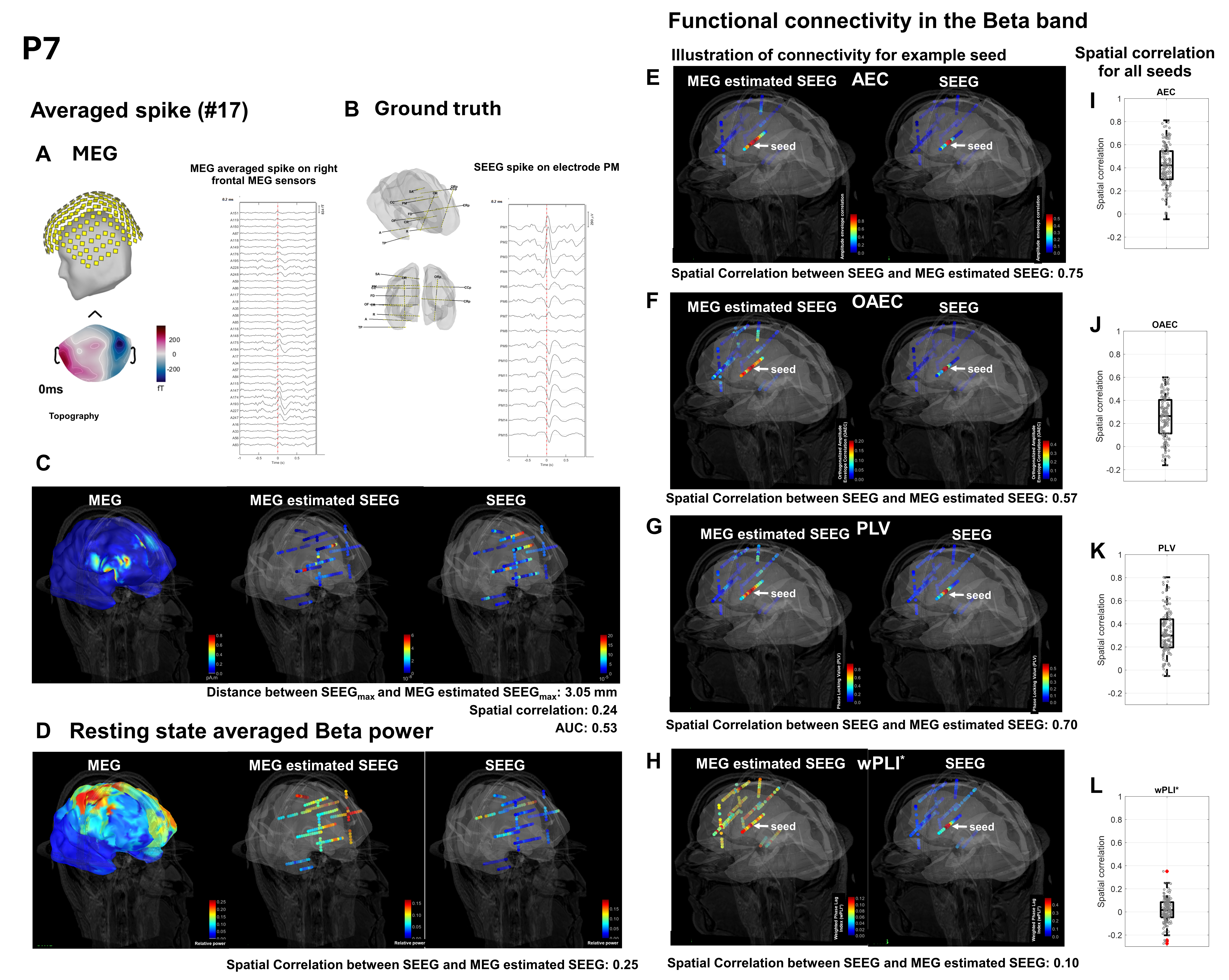


**Fig S5:** Validation of MEG source imaging with simultaneous SEEG for P7. **Spike analysis: (A)** Average of 17 spikes in MEG shown with topography. **(B)** Ground truth: The SEEG implantation and the average of 17 spikes are shown. **(C)** The left panel shows MEG source reconstructed map using cMEM method on the cortical surface at the peak of the spike. The right panel shows the activity of SEEG contacts at the peak of the spike. The middle panel shows the reconstructed source map converted to the SEEG space. The distance between the contact showing maximum activity in SEEG and MEG estimated SEEG is displayed. **Analysis of resting state power in beta band**: **(D)** MEG reconstructed average beta band power is shown on the left panel. The middle panel shows MEG estimated SEEG beta power. The right panel is actual SEEG beta power. The spatial correlation between SEEG and MEG estimated SEEG beta band power is displayed. **Functional connectivity in beta band:** The functional connectivity for an example seed (between the seed and all contacts) is shown for SEEG and MEG estimated SEEG computed using **(E)** Amplitude Envelope Correlation (AEC), **(F)** Orthogonalized Amplitude Envelope Correlation (OAEC), **(G)** Phase Locking Value (PLV) and **(H)** weighted Phase Lag Index (wPLI*). The spatial correlations between SEEG and MEG estimated SEEG for all seeds are summarized as boxplots for **(I)** AEC, **(J)** OAEC, **(K)** PLV and **(L)** wPLI*. Outliers are shown as red dots.


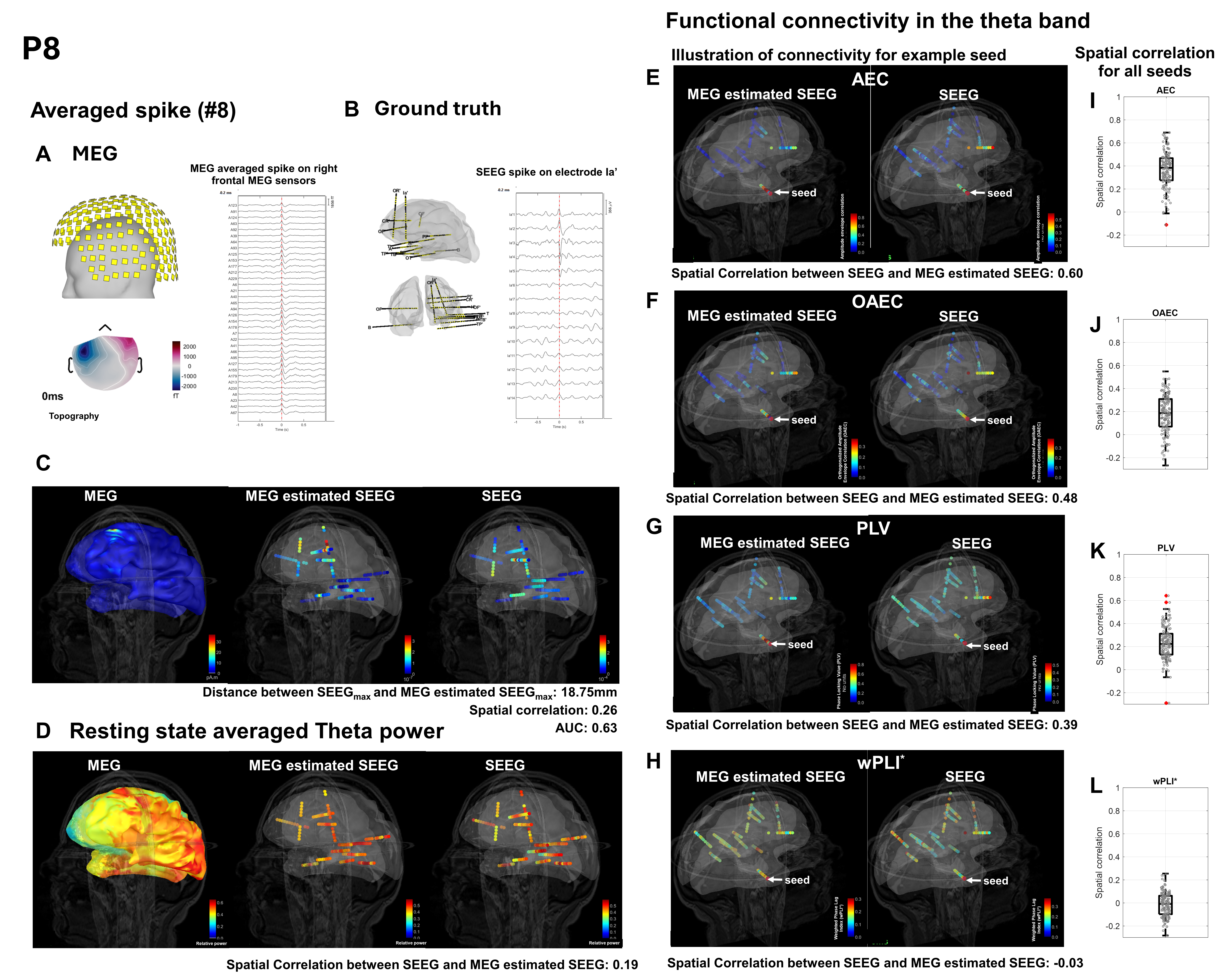


**Fig S6:** Validation of MEG source imaging with simultaneous SEEG for P8. **Spike analysis: (A)** Average of 8 spikes in MEG shown with topography. **(B)** Ground truth: The SEEG implantation and the average of 8 spikes are shown. **(C)** The left panel shows MEG source reconstructed map using cMEM method on the cortical surface at the peak of the spike. The right panel shows the activity of SEEG contacts at the peak of the spike. The middle panel shows the reconstructed source map converted to the SEEG space. The distance between the contact showing maximum activity in SEEG and MEG estimated SEEG is displayed. **Analysis of resting state power in theta band**: **(D)** MEG reconstructed average beta band power is shown on the left panel. The middle panel shows MEG estimated SEEG beta power. The right panel is actual SEEG beta power. The spatial correlation between SEEG and MEG estimated SEEG beta band power is displayed. **Functional connectivity in theta band:** The functional connectivity for an example seed (between the seed and all contacts) is shown for SEEG and MEG estimated SEEG computed using **(E)** Amplitude Envelope Correlation (AEC), **(F)** Orthogonalized Amplitude Envelope Correlation (OAEC), **(G)** Phase Locking Value (PLV) and **(H)** weighted Phase Lag Index (wPLI*). The spatial correlations between SEEG and MEG estimated SEEG for all seeds are summarized as boxplots for **(I)** AEC, **(J)** OAEC, **(K)** PLV and **(L)** wPLI*. Outliers are shown as red dots.


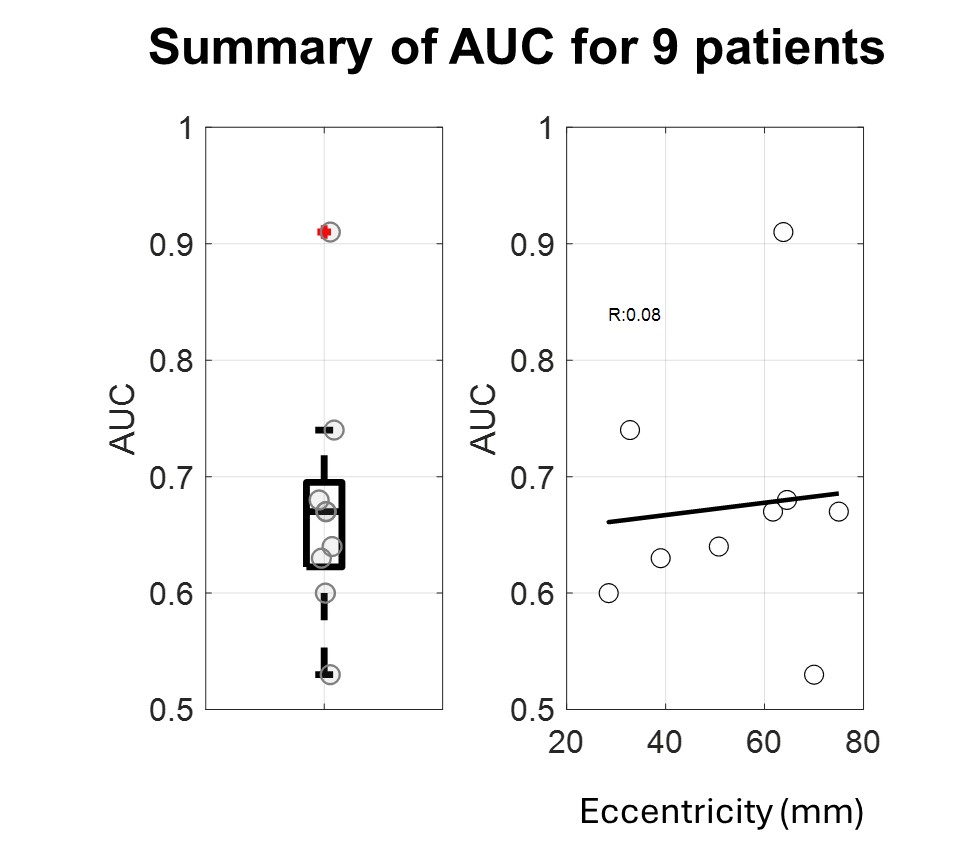


**Fig S7:** Summary of AUC for MEG-estimated SEEG for spike for nine patients. AUC values are summarized as a boxplot distribution (left). AUC as a function of source depth (right), expressed by the eccentricity of the SEEG channel with maximum spike activity. Low eccentricity values correspond to deep sources, and high eccentricity values to superficial sources (Pearson correlation = 0.08, p = 0.8). Outliers are shown as red dots.


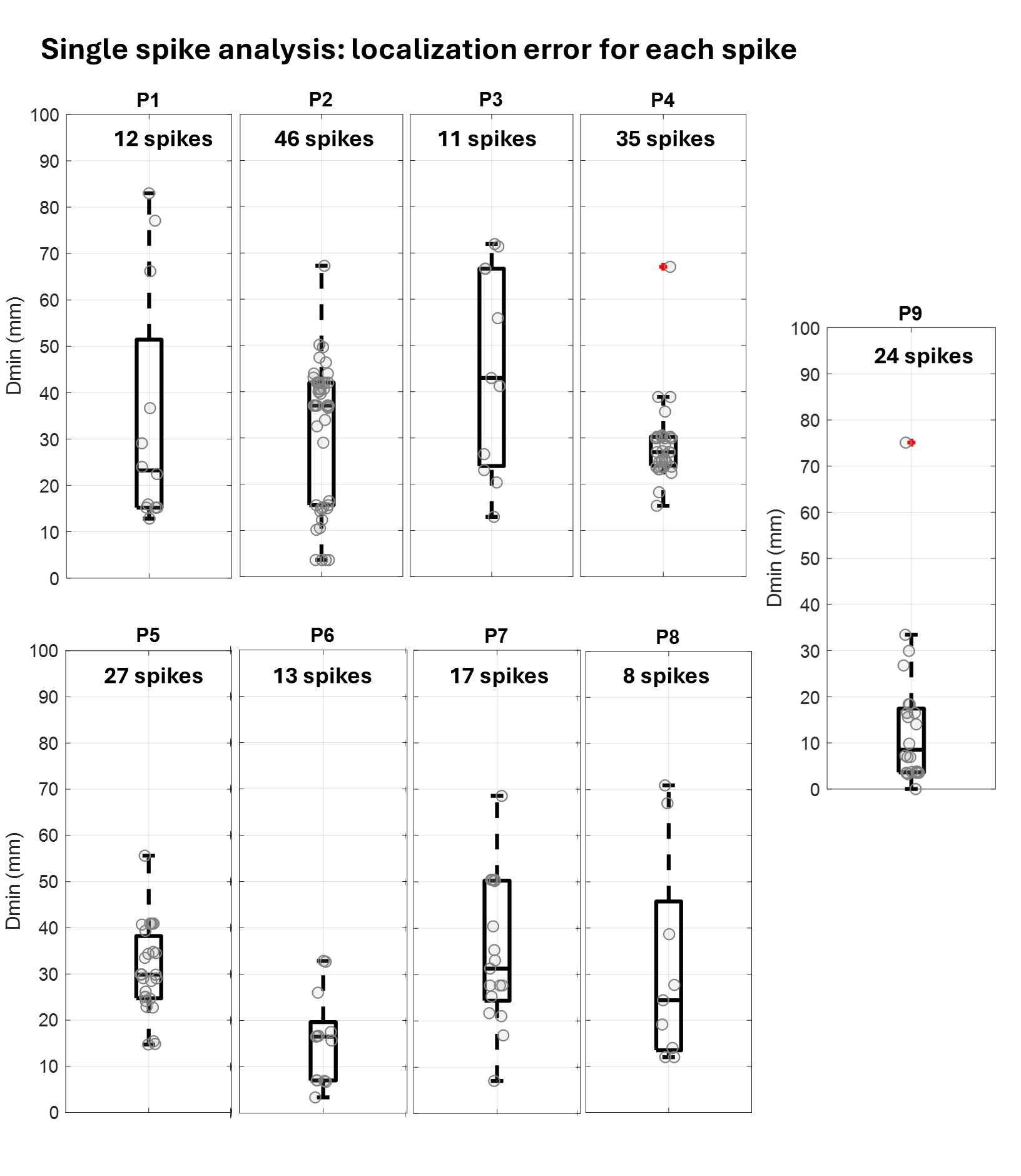


**Fig S8:** Localization errors for single spike for each patient. Outliers are shown as red dots.


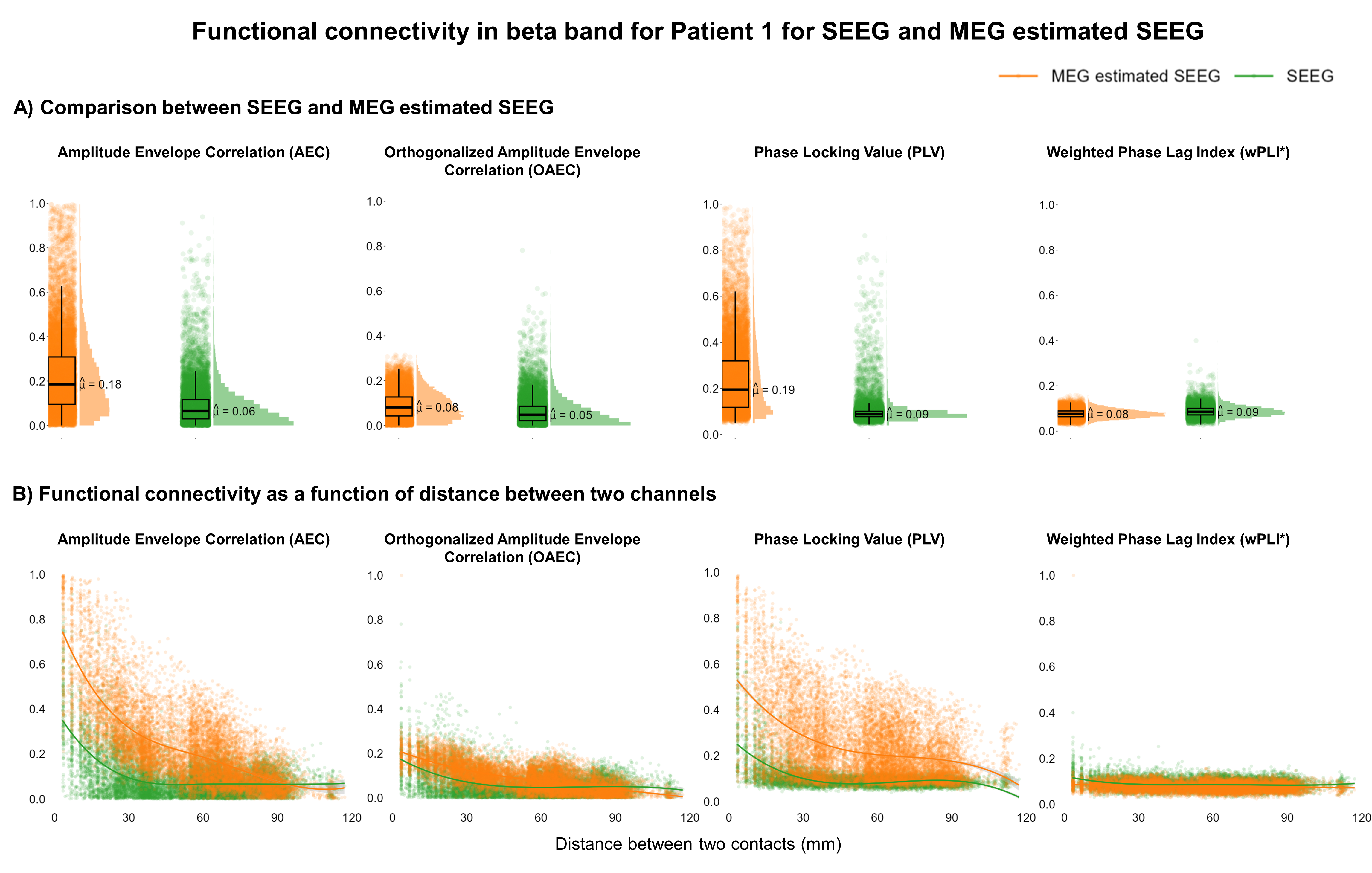


Fig.S9 Resting state functional connectivity values for 13,041 channel pairs estimated by MEG estimated SEEG (orange) and SEEG (green) in Patient 1 (P1) calculated using $\boldsymbol{AEC}$, $\boldsymbol{OAEC}$, $\boldsymbol{PLV}$, and $\boldsymbol{wPLI}^{\boldsymbol{*}}$ in the Beta band (involving a total of 162 channels in bipolar reference montage). (A) In addition to displaying connectivity values for every channel pair (each point on the graph), the distribution of these values is also shown using boxplots, with the median of each distribution also indicated. (B) Functional connectivity between all channel pairs plotted as a function of the distance between channel pairs.

References

1. Cosandier-Rimélé, D., Merlet, I., Badier, J.-M., Chauvel, P. & Wendling, F., 2008. The neuronal sources of EEG: modeling of simultaneous scalp and intracerebral recordings in epilepsy. *NeuroImage* 42, 135-146.

2. Grova, C.*,* Aiguabella, M., Zelmann, R., Lina, J.-M., Hall, J.A. and Kobayashi, E., 2016. Intracranial EEG potentials estimated from MEG sources: A new approach to correlate MEG and iEEG data in epilepsy. *Human brain mapping* 37, 1661-1683.

3. Brookes, M.J., Hale, J.R., Zumer, J.M., Stevenson, C.M., Francis, S.T., Barnes, G.R., Owen, J.P., Morris, P.G. and Nagarajan, S.S., 2011. Measuring functional connectivity using MEG: methodology and comparison with fcMRI. *Neuroimage*, *56*(3), pp.1082-1104.

4. Hipp, J.F., Hawellek, D.J., Corbetta, M., Siegel, M. and Engel, A.K., 2012. Large-scale cortical correlation structure of spontaneous oscillatory activity. *Nature neuroscience*, *15*(6), pp.884-890.

5. Lachaux, J.P., Rodriguez, E., Martinerie, J. and Varela, F.J., 1999. Measuring phase synchrony in brain signals. *Human brain mapping*, *8*(4), pp.194-208.

6. Mormann, F., Lehnertz, K., David, P. and Elger, C.E., 2000. Mean phase coherence as a measure for phase synchronization and its application to the EEG of epilepsy patients. *Physica D: Nonlinear Phenomena*, *144*(3-4), pp.358-369.

7. Bruña, R., Maestú, F. and Pereda, E., 2018. Phase locking value revisited: teaching new tricks to an old dog. *Journal of neural engineering*, *15*(5), p.056011.

8. Vinck, M., Oostenveld, R., Van Wingerden, M., Battaglia, F. and Pennartz, C.M., 2011. An improved index of phase-synchronization for electrophysiological data in the presence of volume-conduction, noise and sample-size bias. *Neuroimage*, *55*(4), pp.1548-1565.
